# Functional genomics reveals an off-target dependency of drug synergy in gastric cancer therapy

**DOI:** 10.1007/s10120-024-01537-y

**Published:** 2024-07-20

**Authors:** Ozen Leylek, Megan E. Honeywell, Michael J. Lee, Michael T. Hemann, Gulnihal Ozcan

**Affiliations:** 1 Koç University Research Center for Translational Medicine, 34450 Istanbul, Turkey; 2 Department of Systems Biology, UMass Chan Medical School, Worcester, MA 01605 USA; 3 Program in Molecular Medicine, UMass Chan Medical School, Worcester, MA 01605 USA; 4 Department of Biology, Massachusetts Institute of Technology, Cambridge, MA 02139 USA; 5 MIT Koch Institute for Integrative Cancer Research, Cambridge, MA 02139 USA; 6 Broad Institute of MIT and Harvard, Cambridge, MA 02139 USA; 7 Department of Medical Pharmacology, Koç University School of Medicine, 34450 Istanbul, Turkey

**Keywords:** Functional genomics, Combination therapy, Synergism, Gastric adenocarcinoma, Genetic dependency

## Abstract

**Background:**

Integrating molecular-targeted agents into combination chemotherapy is transformative for enhancing treatment outcomes in cancer. However, realizing the full potential of this approach requires a clear comprehension of the genetic dependencies underlying drug synergy. While the interactions between conventional chemotherapeutics are well-explored, the interplay of molecular-targeted agents with conventional chemotherapeutics remains a frontier in cancer treatment. Hence, we leveraged a powerful functional genomics approach to decode genomic dependencies that drive synergy in molecular-targeted agent/chemotherapeutic combinations in gastric adenocarcinoma, addressing a critical need in gastric cancer therapy.

**Methods:**

We screened pharmacological interactions between fifteen molecular-targeted agent/conventional chemotherapeutic pairs in gastric adenocarcinoma cells, and examined the genome-scale genetic dependencies of synergy integrating genome-wide CRISPR screening with the shRNA-based signature assay. We validated the synergy in cell death using fluorescence-based and lysis-dependent inference of cell death kinetics assay, and validated the genetic dependencies by single-gene knockout experiments.

**Results:**

Our combination screen identified SN-38/erlotinib as the drug pair with the strongest synergism. Functional genomics assays unveiled a genetic dependency signature of SN-38/erlotinib identical to SN-38. Remarkably, the enhanced cell death with improved kinetics induced by SN-38/erlotinib was attributed to erlotinib’s off-target effect, inhibiting ABCG2, rather than its on-target effect on EGFR.

**Conclusion:**

In the era of precision medicine, where emphasis on primary drug targets prevails, our research challenges this paradigm by showcasing a robust synergy underpinned by an off-target dependency. Further dissection of the intricate genetic dependencies that underlie synergy can pave the way to developing more effective combination strategies in gastric cancer therapy.

**Supplementary Information:**

The online version contains supplementary material available at 10.1007/s10120-024-01537-y.

## Introduction

The evolution of cancer therapy has witnessed a transition toward targeted interventions aimed at key oncogenic drivers and signaling pathways through molecular-targeted agents, offering an attractive strategy to overcome chemoresistance. Departing from traditional regimens involving multiple chemotherapeutics, this strategy leverages molecular-targeted agents distinct modes of action alongside chemotherapeutics, which can address tumor heterogeneity, prevent cross-resistance, and achieve a more tumor-specific activity [1, 2]. However, distinct modes of action do not guarantee a synergistic effect or, at least, an additive effect on cancer cell death [3], underscoring the necessity of selecting combinatorial agents rationally based on a clear understanding of the genetic dependencies and vulnerabilities for synergistic action [4, 5]. Such an understanding also paves the way for discovering novel agents with potent synergy in anti-cancer action.

Functional genomics screens emerge as pivotal tools for unraveling the intricate molecular mechanisms of drug action and pharmacological interactions in combination therapy. These screens have successfully revealed critical dependencies for drug response and chemoresistance successively in cancer cells. Notably, CRISPR screening and clone tracing showed low cross-resistance among the constituents of the R-CHOP (rituximab, cyclophosphamide, adriamycin, oncovin, prednisolone) regimen [6]. A shRNA-based signature assay developed by Pritchard et al. to characterize and predict the mechanisms of drug action enabled dissecting the mode of synergism [5, 7]. This functional assay integrated with informatics tools showed that synergistic chemotherapeutics can exhibit a genetic signature identical to the signature of the dominant chemotherapeutic within the combination, as in the case of 5-FU/Leucovorin, where leucovorin reinforces the action of 5-FU. Whereas some other combinations may exhibit an average of the genetic dependencies of individual chemotherapeutics in the combination, exemplified by actinomycin D/chlorambucil and CHOP. When combined, drug molecules may also reveal a novel genetic signature distinct from their signatures as monotherapy, as suggested in yeast models [8]. In theory, molecular-targeted agents and conventional chemotherapeutics may also interact in line with one of these genetic dependency profiles when combined. However, in the context of targeted agents, understanding the mechanisms underlying drug synergy is particularly challenging, as these drugs can reprogram intracellular signaling pathways, a mode of action distinct from conventional chemotherapeutics. Compounding this complexity is the emerging evidence suggesting that the anti-cancer efficacy of many molecular-targeted agents hinges on off-target effects rather than known primary targets [9]. In light of these challenges, our study focuses on deciphering the genetic dependency signature of synergistic pairs of molecular-targeted agents and conventional chemotherapeutics in gastric adenocarcinoma employing cutting-edge functional genomics approaches and death rate assessment.

Gastric adenocarcinoma, characterized by its intractability and chemoresistance, lacks effective molecular-targeted agents for the majority of patients [10]. Molecular targets, such as epidermal growth factor receptor (EGFR), mammalian target of rapamycin (mTOR), and c-MET, have shown significant involvement in the aggressiveness of gastric adenocarcinomas. Despite the preclinical promise, translating these insights into successful clinical outcomes remains elusive [11–13], possibly due to an irrational combination of EGFR, mTOR, and c-MET targeting agents with chemotherapeutics. Our study addresses this gap by screening pairwise pharmacological interactions of EGFR, mTOR, and c-MET inhibitors with chemotherapeutics from five distinct groups in gastric adenocarcinoma cells, rigorously inspecting the kinetics of cell death. Through a genome-wide CRISPR screen and a shRNA-based signature assay, we aim to unravel the genetic dependency signatures governing the synergy between molecular-targeted agents and chemotherapeutics.

## Materials and methods

### Cell lines and culture conditions

AGS (American Tissue Type Culture Collection, USA), SNU1, SNU5, SNU16, SNU484, and NCI-N87 gastric adenocarcinoma cell lines (Korean Cell Line Bank), and Lenti-X cells were grown in RPMI (Corning, USA) supplemented with 10% fetal bovine serum (FBS) (Gibco, USA) and 1% Penicillin–Streptomycin (P/S) (Gibco, USA). Eμ-Myc Cdkn2a^Arf−/−^ leukemia cell line was grown in B-cell media (BCM) composed of DMEM and IMDM media supplemented with 10% FBS, 1% P/S and 0.1% 2-mercaptoethanol (Gibco, USA). The EGFR, mTOR, and c-Met mutation status data for gastric cancer cells were retrieved from the DepMap portal developed by Broad Institute [14, 15].

### Assessment of pharmacological interactions for drug pairs

We analyzed responses to cisplatin (Sigma-Aldrich C2210000), doxorubicin (Sigma-Aldrich D2975000), 5-fluorouracil (Sigma-Aldrich F0250000), paclitaxel (Sigma-Aldrich Y0000698), SN-38 (Selleckchem S4908), erlotinib (Selleckchem S7786), everolimus (Selleckchem S1120), and JNJ-38877605 (Selleckchem S1114) with MTT (Acros Organics 158,990,050) assay at the initial screening. Briefly, we treated the cells in 96-well cell culture plates (Corning, USA) with at least seven different concentrations of each drug or the drug combinations. After 5 days of incubation, 3 mg/ml MTT was added to each well and incubated for 4 h in the dark. Formazan crystals, the reduced form of MTT, were dissolved with 13.8% SDS solution (Bio-Rad, USA) containing 1% HCl on a shaker overnight at room temperature, and the absorbance was measured at 570 nm by a microplate reader (Synergy H1, Biotek). The relative viability (RV), the ratio of the signal from live cells in each treated group to the control group, was calculated. We plotted each treatment’s sigmoidal dose–response curve (DRC) using four-parameter logistic regression to assess plateau, hill slope, EC_max_, and EC50 in GraphPad Prism7. In the next step, we inferred the fraction-affected (fa) values normalized to 1 from RV. We computed the combination indices (CI) using fa values for each concentration of monotherapy and combinations on the CompuSyn program developed by Chou and Talalay [16]. CI < 1, CI = 1, and CI > 1 denote synergism, additivity, and antagonism, respectively, for drug pairs.

### Fluorescence-based and lysis-dependent inference of cell death kinetics assay

We performed the fluorescence-based and lysis-dependent inference of cell death kinetics (FLICK) assay to investigate drug-induced cell death, as described in Richard et al. [17]. Briefly, we seeded the cells at a density of 2000–2500 cells/well in 96-well plates. The following day, we treated cells with the drug of interest. We used the SYTOX dye (Invitrogen, USA) to indicate dead cells. The fluorescence of SYTOX was monitored throughout the assay using Tecan Spark or Tecan M1000 plate readers to infer the cell death kinetics. At the end of the experiment, we exposed the cells to 0.1% Triton-X (Thermo Scientific, USA) for lysis, from which we deduced the total population size through the SYTOX signal. In addition to experiment plates, we prepared an untreated '*T0*' plate for the lysis of the cells at the beginning of the experiment to determine the initial cell population size. We used the exponential growth modeling of population sizes at the initial and end time points to extrapolate the total population size at intermediate time points. We calculated the size of live populations using dead population sizes represented by the SYTOX signal and whole population sizes. Based on the information on live, dead, and total populations, we evaluated three different pharmacological metrics: fractional viability (FV), lethal fraction (LF), and normalized growth rate inhibition (GR). We provide the equations for each metric below. The code we used to perform curve fitting on MATLAB is available on GitHub (https://github.com/MJLee-Lab) [18].

$${\text{FV}} = \frac{{live_{treated} }}{{live_{treated} + dead_{treated} }}\;\;{\text{LF}} = \frac{{dead_{treated} }}{{live_{treated} + dead_{treated} }}\;\;{\text{GR}} = 2^{ \wedge } \left( {\frac{{\log 2\left( {\frac{{live_{treated} }}{{live_{T0} }}} \right)}}{{\log 2\left( {\frac{{live_{untreated} }}{{live_{T0} }}} \right)}}} \right) - 1$$

### Quantitative immunoblotting

We washed the cell pellets with ice-cold PBS and lysed them in SDS-lysis buffer (50 mM Tris–HCl, 2% SDS, 5% glycerol, 5 mM EDTA, 1 mM NaF, 10 mM β-GP, 1 mM phenylmethylsulphonyl-fluoride, 1 mM Na_3_VO_4_, protease and phosphatase inhibitors). We loaded the lysates at equal protein amounts and concentrations on 4–15% precast TGX gels (Bio-Rad, USA) or 10% hand-poured SDS-PAGE gels. Proteins were transferred to nitrocellulose membranes, then blocked in 1:1 PBS/Intercept Blocking Buffer (IBB, LI-COR Biosciences, USA). Membranes were incubated at 4 °C overnight with 1:1000 diluted EGFR (Cell signaling #2232), p-EGFR (Tyr1173) (Cell signaling #4407), mTOR (Cell signaling #2972), p-mTOR (Ser2448) (Cell signaling #2971), Met (25H2) (Cell signaling #3127), p-Met (Tyr1234/1235) (Cell signaling #3077) primary antibodies. The next day, after washing, membranes were incubated with 1:15,000 diluted secondary antibodies (LI-COR, USA) at room temperature for 1 h. We visualized the immunoblots on the LI-COR Odyssey CLx scanner. Band intensities were quantified using ImageJ and normalized by internal loading control.

### Flow cytometry

Cell pellets were washed in ice-cold PBS, fixed in ice-cold 70% ethanol, and stored at -20 °C. Before cell cycle progression analysis, each sample was washed twice in ice-cold PBS and resuspended in 10% RNase A in PBS. Propidium iodide (final concentration: 0.5 mg/ml) was added to each sample. To assess apoptotic activity, cells were fixed in 4% formaldehyde in PBS at room temperature for 15 min. After washing with cold PBS, samples were fixed in ice-cold 100% methanol and stored at −20 °C. Before analysis, methanol was removed, and pellets were washed twice with PBS containing 0.1% Tween-20 (PBS-T). Each sample was incubated with 1:500 diluted primary cleaved-caspase3 antibody in 1:1 PBS/IBB at room temperature. After 8 h, samples were washed with PBS-T and incubated with primary cleaved-PARP Alexa-647 antibody and goat anti-rabbit Alexa-488 (diluted 1:250 in 1:1 PBS-T/IBB) overnight at room temperature.

For DNA damage analysis, cells were incubated with primary phospho-H2AX antibody (diluted 1:200 in 1:1 PBS-T/IBB) at room temperature for 8 h. Samples were washed with PBS-T and incubated with 1:250 diluted goat anti-mouse Alexa-488 overnight at room temperature. Then samples were washed with PBS and resuspended in 10% RNase A in PBS. Propidium iodide was added to each sample (the final concentration was 0.5 mg/ml). The samples were analyzed in LSRII or Miltenyi MACS Quant VYB flow cytometers. Data analysis was performed on FlowJo.

### Generation of SNU5-Cas9 and SNU5^EGFR−KO^ cells

pCMV-VSV-G (Addgene, USA) [19] and psPAX2 (a gift from Didier Trono; Addgene plasmid #12,260; Addgene, USA) packaging vectors and the equimolar amount of lentiCas9-Blast (Addgene, USA) [20] were diluted in OptiMEM. After adding Lipofectamine 2000 and incubating at room temperature for 15 min, the mixture was added to lenti-X cells. After 6 h, the medium was replaced with a fresh medium. The media with lentiviral particles were collected at 24 and 48 h, then centrifuged and filtered with a 0.45 μm filter. SNU5 parental cells were transduced with viruses containing lentiCas9-Blast by centrifuging at 830xg at 32 °C for 90 min in the presence of polybrene (final concentration: 4ug/ml), which was followed by the replacement with fresh media.

Cells expressing Cas9 (SNU5-Cas9) were selected by blasticidin (10 μg/ml). SgRNA targeting EGFR (5’- CGATCTCCACATCCTGCCGG-3’) was cloned into the lentiGuide-puro vector (Addgene, USA) [20]. SNU5-Cas9 cells were then infected with viruses containing this vector following the protocol described above. SNU5-Cas9 cells expressing sgRNA were selected by puromycin (2 μg/ml). Single-cell clones were generated by seeding cells into 96-well plates, with one cell per well. The EGFR expression in each clone was evaluated via immunoblotting. The Sanger sequencing further confirmed the gene knockout for the selected SNU5EGFR-KO clone.

### Genome-wide CRISPR screen

We performed a genome-wide CRISPR screening using Toronto KnockOut Library v3 (TKOv3) with 71,090 sgRNAs (70,948 sgRNA targeting 18,053 protein-coding genes—4 sgRNA/gene—and 142 control non-targeting sgRNAs against EGFP, LacZ, and luciferase) (Addgene, USA) [21]. More than 300 × 10^6^ SNU5-Cas9 cells were infected with the library to achieve 500 × coverage. After expansion in fresh media, the cells were plated in duplicate 90 × 10^6^ and 50 × 10^6^ cells for each treatment and untreated condition, respectively. 50 × 10^6^ cells were collected and frozen in duplicate for the T0 controls. Cells were treated with either SN-38 (13.5 nM) or the combination (3.7 nM SN-38 + 370 nM Erlotinib) on day 0. On day 2, cells in all experiment groups were passaged by maintaining culture conditions. Live and dead fractions in each group were separated using annexin-V-conjugated magnetic bead sorting on day 3.

Genomic DNA was extracted with the genomic DNA purification kit (Promega, USA). The TKOv3 CRISPR sequencing library was prepared using a two-step PCR reaction: the PCR1 to enrich the gRNA regions in the genome and the PCR2 to amplify gRNAs with sequencing adapters. The 200 bp PCR2 product was excised, and DNA was purified from agarose gel with a gel extraction kit (Qiagen, Germany). Reads of each sequenced library were demultiplexed using the barcode splitter and trimmer functions of the FASTX toolkit. Reads were mapped to the TKOv3 library by the Bowtie2 read alignment tool. A parametric fit of DESeq2 was used to determine the sgRNA-level log2 fold change (L2FC) for each comparison. Then we randomly assigned the non-targeting sgRNA controls to 36 sets of four sgRNAs per gene. Guide-level scores were transformed into the single-gene-level fold change (FC) by calculating the mean of all sgRNAs. FCs at the gene level were *z*-scored based on non-targeting genes’ mean and standard deviation. Empiric *p *values were calculated from *z*-scored FCs by bootstrapping guide-level scores, which were then false discovery rate (FDR)-corrected with the Benjamini–Hochberg procedure.

### CRISPR screen validation

All sgRNAs targeting selected candidate hits and non-targeting control sgRNA targeting the LacZ gene were cloned in the pRDA_170 vector with the e2-crimson dark red fluorescence protein sequence. SNU5-Cas9 cells were transduced with viruses containing corresponding vectors to generate the knockout bulk populations (SNU5^*Gene X*−KO^) and the untargeted control population (SNU5^LacZ^), followed by puromycin selection.

Two different approaches were adopted for the validation, and the treatments were performed at doses applied in the genomic screening. The first was done by FLICK assay, as explained above. The second validation was performed using a competition assay. For this, SNU5-Cas9 and SNU5-Cas9 cells with sgRNA targeting gene of interest were mixed at a 1:1 ratio and seeded in 12-well plates (2 × 10^5^ cells/well). The enrichment/depletion of e2-crimson-positive cells was assessed after 3 days of drug treatment using BD FACS Celesta flow cytometry. Data analysis was performed on FlowJo.

### RNAi-based signature assay

Eight shRNAs in pMSCV-LTR-miR30-SV40-GFP (MLS) retroviral vector, validated for their knock-down and off-target effects, were used for signature assay [5, 7]. Eμ-Myc Cdkn2a^Arf−/−^ cells were transduced with each GFP-tagged shRNA construct at a 25–30% infection rate. Cells were seeded into 24-well plates in 250 μl BCM and treated with 250 μl of drug-containing media, inducing 80–90% cell death (LD80-90) at 48 h. At the endpoint, the live cells were examined by DAPI exclusion, and the percentage of GFP-positive cells was determined using BD FACS Celesta flow cytometry. Resistance index (RI), calculated following the formula: (GFPtreatment—(GFPtreatment* GFPcontrol))/ (GFPcontrol—(GFPtreatment* GFPcontrol)), was used to generate the signature of each drug of interest. Each signature was then compared with a previously established reference set of drugs using the modified Euclidean *K*-nearest neighbors algorithm via MATLAB, generating linkage ratios (LR) and *p* values [22]. An LR ≤ 1 and a *p* value < 0.05 indicate a significant classification of the drug of interest into a specific category in the reference set. LR and/or *p* values outside of these cutoffs are interpreted as the drug of interest that belongs to a “new class” with a mechanism of action not represented in the reference set.

### qRT-PCR

RNA was extracted from 1–1.5 × 10^6^ cells using the Bio-Rad RNA extraction kit (USA). The RNA was combined with 2.5 μl of 2 nM dNTPs (Life Technologies, USA) and 1 μl of random hexamers (Invitrogen, USA) per sample for cDNA synthesis. After incubation at 65 °C for 5 min, an RT mix of 5 μl first-strand buffer (Invitrogen, USA), 2 μl of 0.1 M DTT (Invitrogen, USA), and 0.5 μl of Rnasin (Promega, USA) were added to each tube and incubated at room temperature for 10 min. Finally, 1 μl of MMLV-RT enzyme (Invitrogen, USA) was added. The reactions were incubated at 37 °C for 1 h and then inactivated at 70 °C for 15 min. For the qRT-PCR reaction, 10 μl of 2X Fast SYBR Green Master Mix (Applied Biosystems, USA), 1 μl of 4 μM primer mix (ABCG2-F: AGCCACAGAGATCATAGAGCC, ABCG2-R: TTCTCACCCCCGGAAAGTTG, GAPDH-F: AGCCACATCGCTCAGACAC, GAPDH-R: GCCCAATACGACCAAATCC), and 7 μl nuclease-free grade water were mixed with 2 μl of diluted cDNA sample in each well of a 96-well optical plate (Applied Biosystems, USA). The CT values for each reaction were determined using the StepOnePlus Real-Time PCR System (Applied Biosystems). The GAPDH housekeeping gene was used to calculate the relative expression level of ABCG2.

### Assessment of efflux pump activity

SNU5 cells seeded in 12-well plates were treated with erlotinib, osimertinib, and ABCG2i-1, ABCG2i-2 at 10, 1, 0.1, and 0.01 μM. The following day, cells were exposed to Hoechst-33342 (final concentration: 5 μg/mL) for 45 min at 37 °C in the dark. Cells were washed with cold Hanks balanced saline solution (Gibco, USA) with 1 mM HEPES (Gibco, USA) and 2% FBS (HBSS+), and then taken into fresh media. Hoechst-33342 negativity was evaluated at times 0, 1 h, and 16 h using the FACSymphony A3 flow cytometer (BD Biosciences, USA) by analyzing at least 10,000 events per sample. Data analyses were performed on FlowJo.

### Analysis of pan-cancer cell line and colorectal cancer patient data

To investigate the effect of SLFN11 expression in relation to ABCG2 expression on the sensitivity to SN-38, we downloaded gene expression and drug sensitivity data of 411 pan-cancer cell lines from DepMap Portal (https://depmap.org/portal/) [14, 15]. The medians of SLFN11 and ABCG2 expression among 411 cell lines were used as a cutoff to classify cell lines as SLFN11 and ABCG2 high or low. The Mann–Whitney test was used to compare groups. The data for colorectal cancer patients were downloaded from RocPlotter (https://rocplot.org/) [23]. The CI of the SN-38/gefitinib combination in HT-29 cells was retrieved from Azzariti et al. 2004 [24], and in COLO320 DM, Lovo, and SW480 cells, the CI values were retrieved from Kouzimu et al. 2004 [24, 25]. The CI values at fa = 0.5 (fraction of affected cells = 0.5) were used to investigate the correlation between drug synergy and ABCG2 expression levels of the cell lines downloaded from merav.wi.mit.edu/ [26].

## Results

### Identifying synergistic molecular-targeted agent and conventional chemotherapeutic pairs in gastric adenocarcinoma cells

**Fig. 1:**
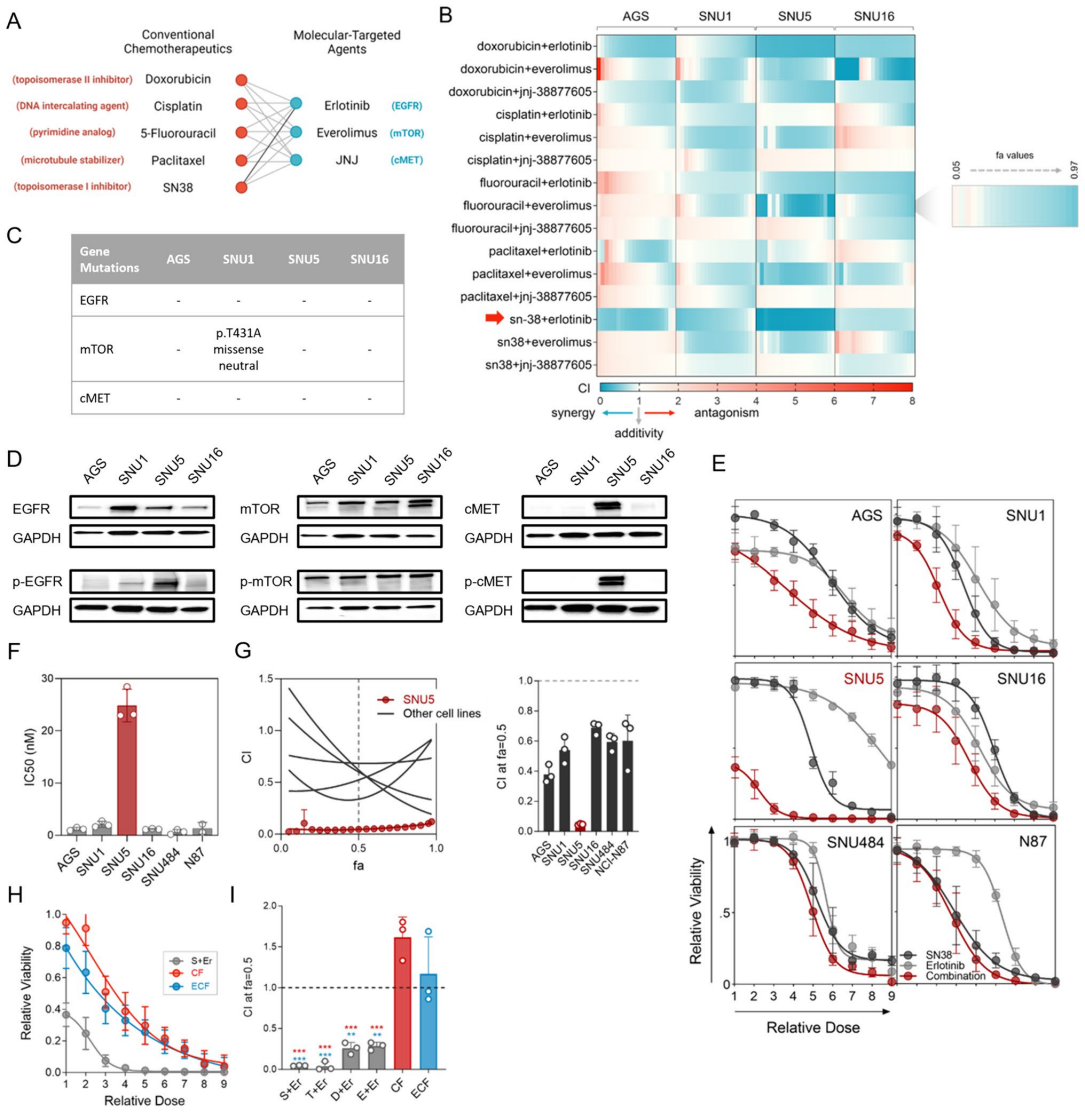
Pharmacological interactions for the molecular-targeted agent and conventional chemotherapeutic combinations in gastric adenocarcinoma cells. **A** The molecular-targeted agent and conventional chemotherapeutic pairs assessed in this study. The figure was generated on BioRender. **B** Heatmap of combination indices (CI) at fraction affected (fa) values ranging from 0.05–0.97 for each drug pair in AGS, SNU1, SNU5, and SNU16 cells calculated by Chou–Talalay’s method. Blue indicates synergism, and white and red indicate additivity and antagonism. The strongest synergism was observed for the SN-38/erlotinib pair in SNU5 cells (highlighted by a red arrow). **C** The mutation status of EGFR, mTOR, and c-MET in gastric adenocarcinoma cells. **D** The expression and basal phosphorylation of EGFR, mTOR, and c-MET in gastric adenocarcinoma cells. **E** Dose–response curves for SN-38, erlotinib, and SN-38/erlotinib combination in AGS, SNU1, SNU5, SNU16, SNU484, and NCI-N87 gastric adenocarcinoma cells. **F** IC_50_ values for SN-38 in all cell lines. **G** CI-fa plots generated using the data in E. **H** Dose–response curves for SN-38/erlotinib, cisplatin–5-fluorouracil (CF), and epirubicin–cisplatin–fluorouracil (ECF) combinations in SNU5 cells. **I** The CIs for the combination of erlotinib with topoisomerase poisons (SN-38, topotecan, epirubicin, and doxorubicin), CF, and ECF regimens (left). The CI plots were generated using the data in E, H, and Fig. S1-C and D. Dunnett’s multiple comparisons test was used for statistical analysis. ***p < 0.001, **p = 0.001

To identify molecular-targeted agent–conventional chemotherapeutic pairs with strong synergism, we combined EGFR inhibitor erlotinib, mTOR inhibitor everolimus, and c-MET inhibitor JNJ-38877605 with chemotherapeutics from five distinct groups acting via diverse mechanisms: doxorubicin (anthracycline), cisplatin (platinum derivative), 5-fluorouracil (fluoropyrimidine), paclitaxel (taxane), and SN-38 (topoisomerase I inhibitor) (Fig. 1A). We investigated the pharmacological interactions for these 15 drug pairs in AGS, SNU1, SNU5, and SNU16 gastric adenocarcinoma cell lines using Chou–Talalay’s CI method (Fig. 1B, Fig. S1A) [16]. These cell lines do not carry any loss or gain of function mutations, translocations, or fusions involving EGFR, mTOR, or c-MET. Only SNU1 cells carry a missense variation (p.T431A) in mTOR which does not affect mTOR function (Fig. 1C). The cell lines exhibited different EGFR, mTOR, and c-MET expression levels and basal phosphorylation (Fig. 1D). Hence, we tested the drug pairs in gastric cancer cells with varying target expression and phosphorylation profiles.

In the pairwise pharmacological interaction screen, erlotinib and SN-38 (the active metabolites of irinotecan) emerged as the most synergistic combination (Fig. 1B). The other drug pairs exhibited antagonism, additivity, or weaker synergy compared to erlotinib/SN-38. Since these drug pairs do not provide robust models for strong synergism and are unlikely or less likely to be effective in cancer therapy, we have not pursued further investigation into the molecular mechanisms of these interactions and focused on the SN-38/erlotinib combination.

The potency of the SN-38/erlotinib combination was significantly higher than that of SN-38 and erlotinib as single agents in six different gastric adenocarcinoma cell lines (Fig. 1E). Among these cell lines, SNU5 cells, derived from the metastatic ascites fluid of a poorly differentiated gastric adenocarcinoma patient who had previously undergone chemotherapy [27], exhibited the lowest sensitivity to SN-38 (Fig. 1F). Despite that, the synergism for the SN-38/erlotinib combination was the strongest in this cell line (Fig. 1G, Figure S1B). The SN-38/erlotinib combination was much more potent than the cisplatin/5-FU (CF) and epirubicin/cisplatin/5-FU (ECF) combinations, commonly used chemotherapy regimens in gastric cancer therapy (Fig. 1H) [28].

To interrogate whether the synergistic interaction of erlotinib with SN-38 is unique to SN-38 or shared by other topoisomerase inhibitors, we tested its combination with another topoisomerase I inhibitor, topotecan, and topoisomerase II inhibitors, doxorubicin and epirubicin in SNU5 cells (Fig. 1I, Fig. S1C–D). The combination of erlotinib with all topoisomerase poisons exhibited robust synergism in contrast to the antagonistic interaction in CF and ECF. However, the degree of synergism was highest for SN-38/erlotinib combination.

### Validating the synergy in SN-38/erlotinib combination dependent on cell death

**Fig. 2:**
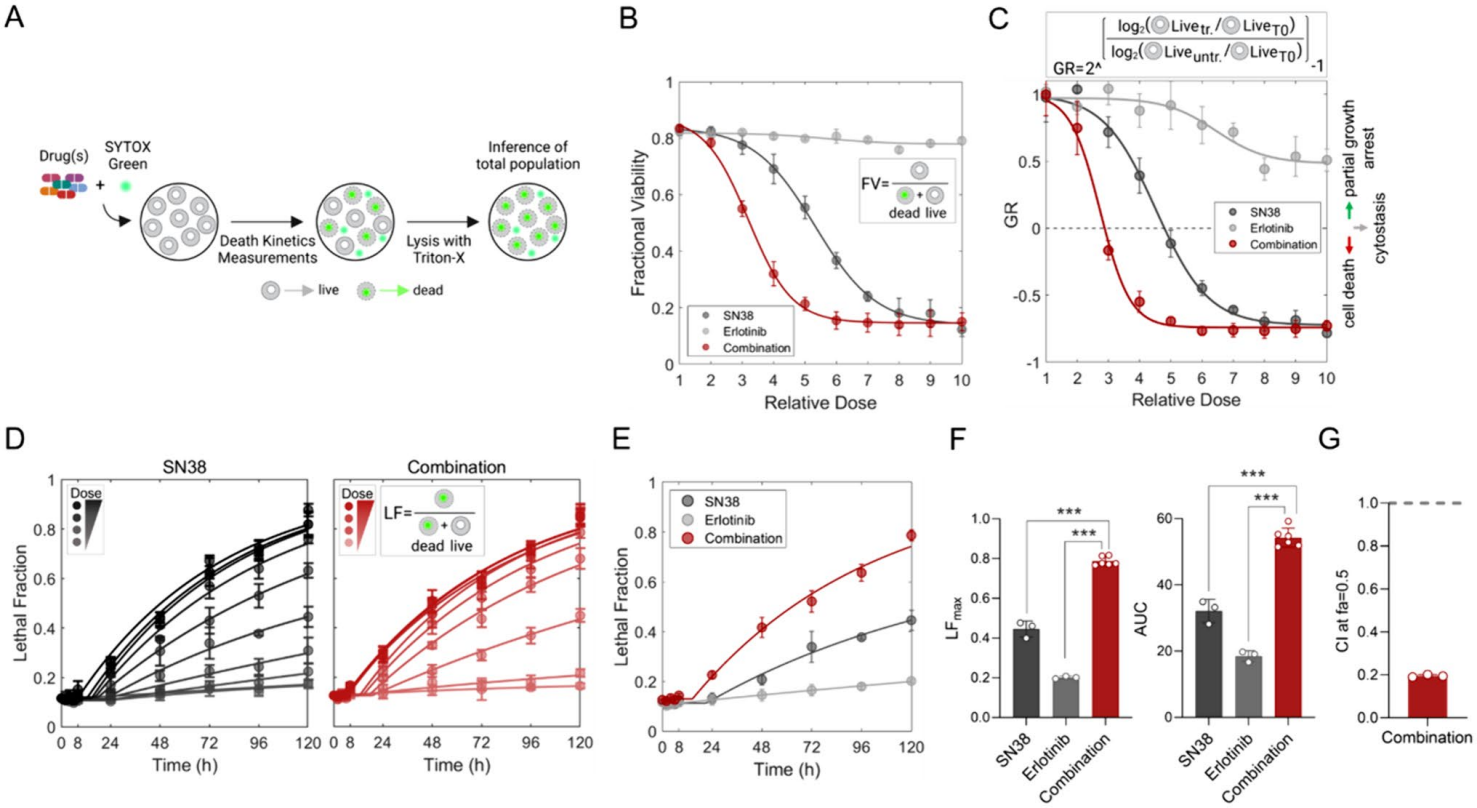
Validating the synergistic action of erlotinib-SN-38 with the fluorescence-based and lysis-dependent inference of cell death kinetics (FLICK) assay. **A** The workflow for the FLICK assay adapted from Honeywell et. al. [64], created in BioRender. **B** Fractional viability (FV), **C** growth rate (GR), and **D** lethal fraction (LF) kinetics of the SN-38/erlotinib combination and single-agent treatments. **B–D** Relative doses are given in Fig. S2A. **C** Negative GR values indicate cell death and positive GR values indicate partial growth arrest. *Untr* untreated, *tr* treated. **E** Comparison of the LF curves for each treatment at relative dose 5. LF curves at all the relative doses are presented in Fig. S2C. **F** Quantifying LF maxima (LF_max_) and area under the curve (AUC) values of data presented in E. Statistical analysis was performed using Dunnett’s multiple comparisons test. ***p < 0.001. **G** CI at fa: 0.5, calculated based on LF metric

The action of drugs in cell metabolism-based screening tests may depend on partial growth inhibition and cytostasis, in addition to cytotoxicity [29]. To determine if the synergistic action of the SN-38/erlotinib combination is due to increased cell death or decreased proliferation rate, we used the FLICK assay (Fig. 2A) [30].

The degree of cell death was evaluated using the fractional viability (FV) metric, which is simply the fraction of the total live cell population. FV analysis showed that the high potency achieved by the SN-38/erlotinib combination was due to enhanced cell death (Fig. 2B, Fig. S2A). To determine if the levels of death observed were sufficient to shrink a tumor population, we also evaluated the normalized growth rate inhibition value (GR value). Negative GR values revealed that the SN-38/erlotinib combination induced a significant shrinkage in population size, indicating high cell death (Fig. 2C). In contrast, as indicated by positive GR values, erlotinib alone caused only a partial growth arrest, whereas SN-38 led to partial growth arrest at low doses and cell death at high doses. We also evaluated the lethal fraction (LF) over time using FLICK to gain further insight into the kinetics of the death induced by these drugs. The SN-38/erlotinib combination triggered cell death more robustly than single-agent treatments (Fig. 2D–F, Fig. S2B–C). CI analysis based on the LF metric revealed a strong synergism (Fig. 2G), similar to CI values inferred from the RV-based analysis (Fig. 1E). Hence, the LF-based assessment further validated that synergism in the SN-38/erlotinib combination relied on an increased cell death rate.

**Fig. 3:**
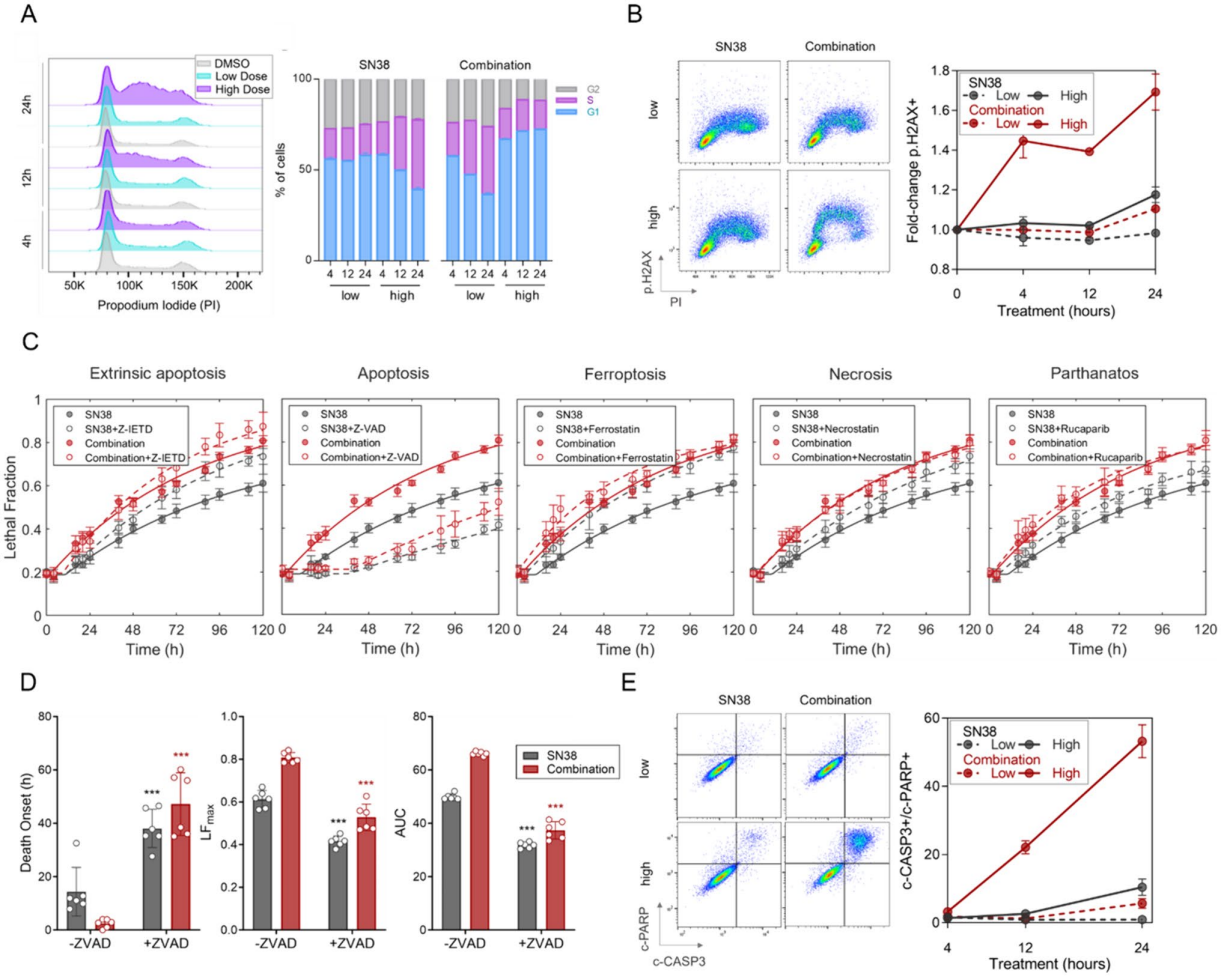
Phenotypic responses to SN-38 alone and SN-38/erlotinib combination in SNU5 cells. **A** Representative histogram for cell cycle progression (left), and percentage of cells at each phase (right), **B** representative flow cytometry plots (left), and fold change in p.H2AX (right) to assess alterations in DNA damage over time under SN-38 or SN-38/erlotinib combination treatment at low or high doses. PI: propidium iodide. p.H2AX: phospho-H2AX. **C** Identification of cell death type triggered by SN-38 alone (30 nM) and SN-38/erlotinib combination (30 nM SN-38 + 3 μM erlotinib). Lethal fraction (LF) kinetics were analyzed over time under both treatments in the presence and absence of indicated inhibitors targeting specified cell death pathways. **D** The death onset time, LF maxima, and AUC parameters of LF curves reporting changes in cell death rate in the presence of apoptosis inhibitor Z-VAD-FMK in C. Statistical analysis was performed using Welch’s *t *test. ***p < 0.001. **E** Analysis of apoptotic response to SN-38 alone or SN-38/erlotinib combination treatment at low and high doses via cleaved-caspase3 (c-CASP3) and cleaved-PARP (c-PARP) co-staining. Representative flow cytometry plots (left) and the kinetics of apoptotic cell death quantified (right)

To investigate growth arrest in detail, we analyzed the cell cycle progression under SN-38 alone or SN-38/erlotinib combination at relatively low and high doses. The low-dose SN-38/erlotinib combination elicited a cell cycle arrest in S-phase, also observed for high-dose SN-38 alone (Fig. 3A, Fig. S3A). However, the high-dose SN-38/erlotinib combination induced a potent cell cycle arrest in the G1 phase and increased DNA damage even at earlier time points (Fig. 3A–B, Fig. S3A–B). Next, we explored the mode of synergistic cell death by investigating the alteration in cell death kinetics in the presence of extrinsic apoptosis, apoptosis, ferroptosis, necrosis, or parthanatos inhibitors (Fig. 3C). Apoptosis inhibitor Z-VAD-FMK substantially decreased the LF under both SN-38 and the SN-38/erlotinib combination treatments, with a significantly delayed death onset (Fig. 3C–D). The incapability of the extrinsic apoptosis-specific inhibitor Z-IETD-FMK to induce a similar change indicated that the cell death by the SN-38/erlotinib combination was through intrinsic apoptosis. The assessment of cleaved-caspase3 and cleaved-PARP further confirmed prominent apoptotic cell death (Fig. 3E, Figure S3C).

### The role of EGFR in synergistic action

**Fig. 4:**
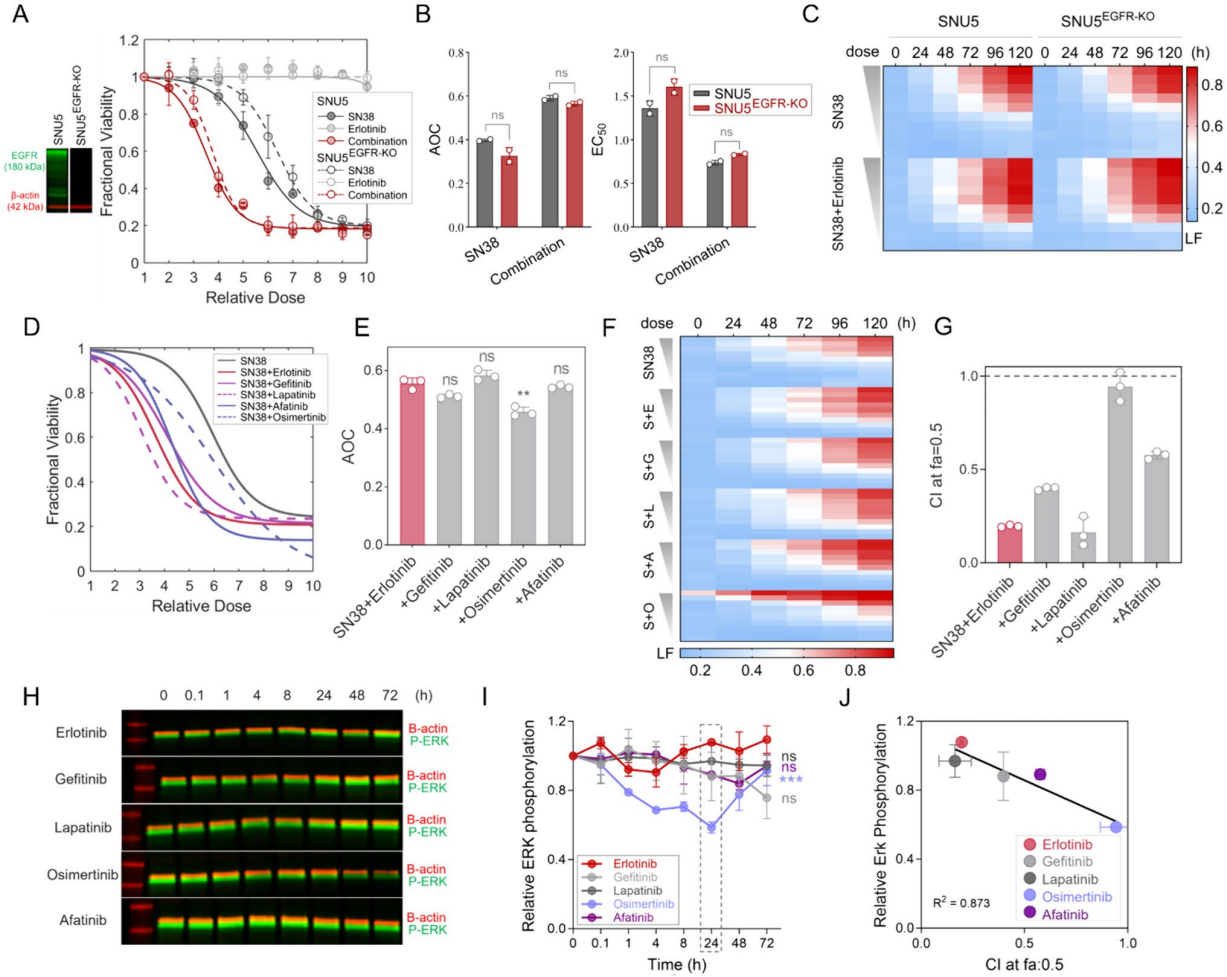
EGFR is not involved in synergistic action. **A** Drug response to the SN-38/erlotinib combination and single agents in SNU^EGFR−KO^ cells compared to SNU5 cells. Immunoblot (left) shows the lack of EGFR expression in SNU^EGFR−KO^ cells. **B** Comparison of the area over the curve (AOC) and EC_50_ parameters deduced from fractional viability (FV) curves in A. Welch’s *t* test was used for statistical analysis. *ns* non-significant. **C** Dose-dependent cell death kinetics over time in SNU^EGFR−KO^ and SNU5 cells. **D** Drug response to SN-38 and its dual combination with EGFR inhibitors. **E** Comparison of the AOC values deduced from FV curves in D, using Dunnett’s multiple comparisons test. **p value = 0.002. **F** Dose-dependent cell death kinetics over time under SN-38 alone and its dual combination with EGFR inhibitors. (*S* SN-38, *E* erlotinib, *G* gefitinib, *L* lapatinib, *O*: osimertinib, *A* afatinib). **G** CI values at fa: 0.5 deduced from LF analysis in F. **H** Immunoblot analysis of time-dependent alterations in ERK activity under treatment with EGFR inhibitors. **I** Densitometric quantitation of immunoblot data in H. The dashed box indicates the time point where a significant decrease in ERK phosphorylation was observed under osimertinib treatment but not other EGFR inhibitors, in comparison with erlotinib. Statistical significance was determined using Dunnett’s multiple comparisons test. ***p < 0.001. **J** Correlation between the level of ERK phosphorylation at 24 h after treatment with the specified EGFR inhibitor and CI value at fa: 0.5 for dual combinations of SN-38 with EGFR inhibitors

To investigate the dependency of synergism on the inhibition of EGFR, the primary target of erlotinib, we analyzed the fractional viability of SNU5^EGFR−KO^ cells in response to SN-38, erlotinib, and SN-38/erlotinib combination (Fig. 4A, B), and the kinetics of cell death via LF metric (Fig. 4C). Unexpectedly, we did not observe a reversal of the synergism elicited by the SN-38/erlotinib combination in SNU5^EGFR−KO^ cells compared to SNU5 parental cells, as FV in SNU5^EGFR−KO^ cells was indifferent from SNU5 cells under each treatment condition (Fig. 4A–B). The evaluation of the LF also revealed that the SN-38/erlotinib combination elicited a similar cell death rate and kinetics in both SNU5^EGFR−KO^ and SNU5 cells (Fig. 4C). These results suggested a mechanism of synergism independent of EGFR inhibition.

Several studies report different efficacies and toxicity profiles for distinct EGFR inhibitors in cancer patients [31]. To understand whether the synergistic interaction of erlotinib with SN-38 is common to different EGFR inhibitors, we tested the combinations of gefitinib, lapatinib, osimertinib, and afatinib with SN-38 in SNU5 cells (Fig. 4D–F, Fig. S4A). The dual combination of SN-38 with all EGFR inhibitors improved the drug response and enhanced the cell death rate compared to SN-38 alone, as in the SN-38/erlotinib combination, except for osimertinib. CI analysis showed that all EGFR inhibitors, but not osimertinib, exhibited synergism with SN-38 (Fig. 4G). To assess whether EGFR inhibitors’ action on other receptor tyrosine kinases (RTK) may be involved in synergism, we examined the kinetics of ERK phosphorylation, a common intracellular target of the RTK family [32] (Fig. 4H–I, Fig. S4B). Except for osimertinib, the EGFR inhibitors did not decrease ERK phosphorylation in SNU5 cells, ruling out the involvement of other RTKs as off-targets of EGFR inhibitors in synergistic response. Surprisingly, the degree of synergism with SN-38 was inversely correlated with the efficacy of EGFR inhibitors to inhibit ERK phosphorylation (Fig. 4J). Since EGFR or RTK signaling through p-ERK (phosphorylated ERK) is not altered by EGFR inhibitors that elicited synergism with SN-38, these findings strengthened that the synergism likely does not result from the loss of EGFR or RTK signaling.

### Investigating the genetic dependency/vulnerability signature for drug synergy with whole-genome CRISPR screening

**Fig. 5:**
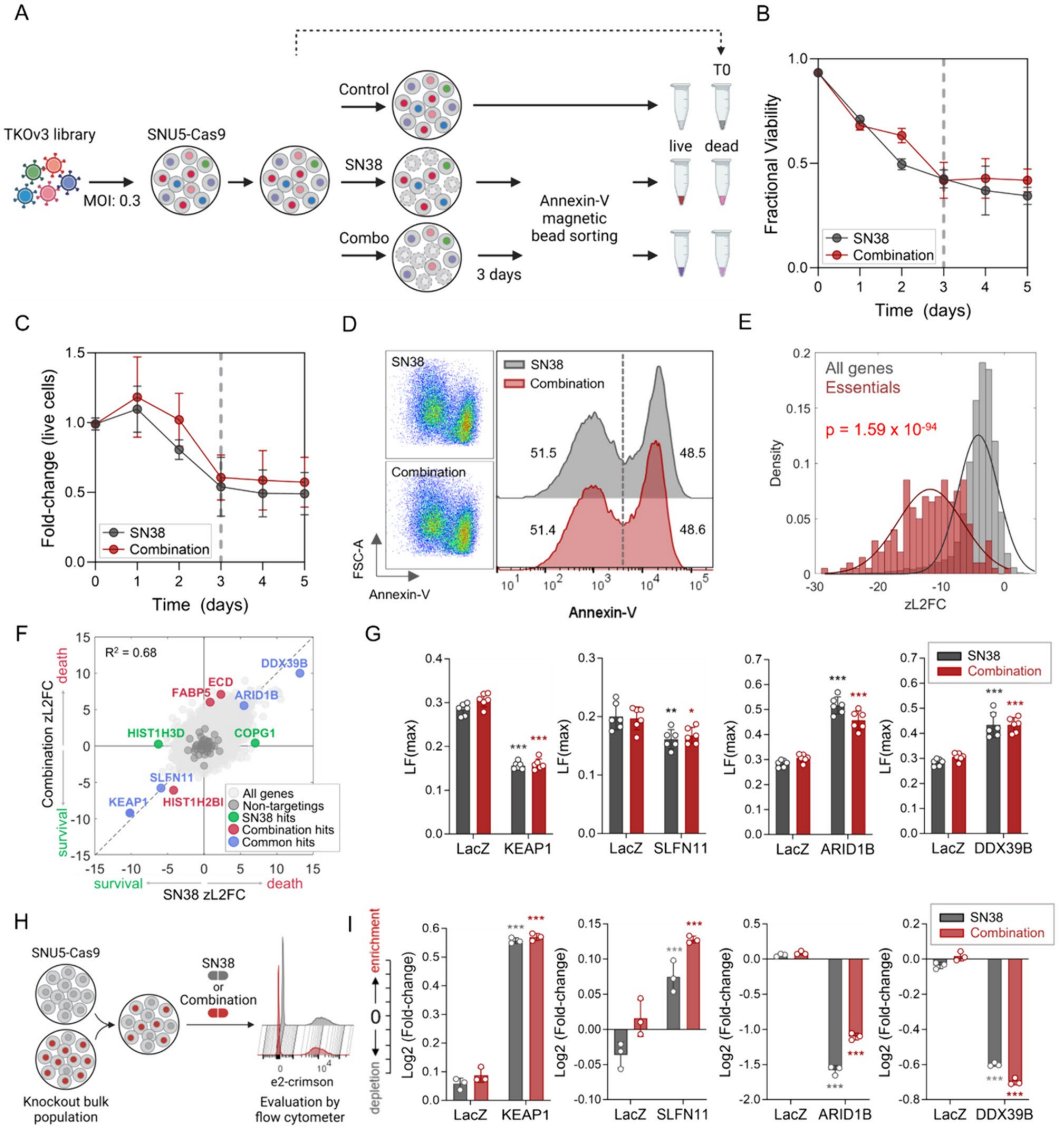
Genome-wide CRISPR screen to explore the mechanism of synergism. **A** Experimental workflow of the genome-wide CRISPR screening coupled with annexin-V magnetic bead sorting. The figure was generated using BioRender. **B–C** Optimization of SN-38 (13.5 nM) and the SN-38/erlotinib combination (3.7 nM SN-38 + 370 nM erlotinib) concentrations and drug exposure period for CRISPR screen based on trypan-blue exclusion. Treatment with SN-38 alone and the SN-38/erlotinib at indicated concentrations for 3 days resulted in (**B**) fractional viability of ~ 0.5 obtained by counting both the number of live and dead cells and (**C**) a ~ 0.5-fold change in the number of live cells in the treated group compared to the untreated. **D** Evaluation of annexin-V positivity under SN-38 alone and the SN-38/erlotinib combination applied at optimized concentrations for 3 days. The assay conditions for both treatments achieved ~ %50 annexin positivity. **E** Distribution of all genes compared to core essential genes in untreated vs. T0 sample at the gene-level zL2FC. The Kolmogorov–Smirnov test was used to calculate the *p *value. **F** Identification of candidate hit genes with an FDR < 0.1 for both SN-38 alone and the SN-38/erlotinib combination treatments, comparing the dead vs. the live populations at the gene-level zL2FC. The knockout of genes highlighted significantly altered cell death or survival rates under SN-38 alone and/or the SN-38/erlotinib. Positive zL2FC values indicate an increase in death rate, and negative zL2FC values indicate an increase in survival rate. The relationship between the two data sets was determined by computing *R*-squared for the genes with an FDR < 0.1. **G** Investigating alterations in the death rate within the knockout populations compared to the untargeted population (LacZ) under SN-38 and SN-38/erlotinib combination by analyzing LF_max_. **H** The schematic representation of the competition assay generated using BioRender. **I** Results of competition assay showing the enrichment/depletion of the knockout populations compared to the untargeted population under SN-38 and SN-38/erlotinib combination. The treatments were applied at the screening doses for 3 days in (**G**) and (**I**). Dunnett’s multiple comparisons test was used to compare the knockout and untargeted populations for each treatment. ***p < 0.001, **0.002, *0.033

To elucidate the distinct genetic dependencies underlying cell death activation via the SN-38/erlotinib combination compared to SN-38 alone, we adopted a comprehensive approach, integrating genome-wide CRISPR screening with annexin-V magnetic bead sorting (Fig. 5A). As shown in Figs. 2E and 3E, SN-38/erlotinib combination induces cell death more potently than SN-38 alone when we apply SN-38 at equimolar concentrations in both treatments. However, in equimolar concentrations, it is hard to discern whether the SN-38/erlotinib combination employs distinct mechanisms of action than SN-38 alone or if erlotinib’s presence reinforces the exact mechanisms of action triggered by SN-38 for cell death. Therefore, we identified equipotent concentrations of SN-38 and SN-38/erlotinib combination that achieve an intermediate level of cell death at a shorter drug exposure period, as opposed to previous CRISPR-based perturbation screens with DNA-damaging agents [33, 34]. Such adjustments ensured that the population size that would guarantee sgRNA representation at > 300 coverage could be maintained throughout the screen, and the sgRNA distribution of the library would mainly be affected by the alterations in cell death rate instead of the alterations in proliferation rate. Exposure to 13.5 nM SN-38 and the combination of 3.7 nM SN-38 and 370 nM erlotinib for 3 days induced ~ %50 annexin-V positivity (Fig. 5B–D). We applied these assay conditions in our pooled screen to unveil the mechanistic traits intrinsic to cell death triggered by SN-38 and SN-38/erlotinib combination.

The quality assessment of the screen data revealed a significant decrease in core essential genes [35] in the untreated vs. T0 sample and a strong correlation between replicates (Fig. 5E, Fig. S5A). We followed a CRISPR death screen approach [36], using the comparison of “dead vs. live” at the gene-level z-scored log_2_ fold change (zL2FC) for the SN-38/erlotinib combination compared to SN-38 to identify differentially enriched or depleted genes under either of these treatments and both (Fig. 5F). The gene-level knockouts significantly enriched/depleted with an FDR < 0.1 in the SN-38/erlotinib combination showed a strong correlation with those in SN-38 alone. The evaluation of the LF metric under SN-38 alone and the SN-38/erlotinib combination demonstrated that deleting KEAP1 or SLFN11 decreased the death rate. In contrast, deleting ARID1B or DDX39B enhanced the death rate compared to the untargeted population, validating the screen results (Fig. 5G).

We performed competition assay in knockout populations to provide insight into the altered cell survival in response to treatments. We observed the enrichment of KEAP1 or SLFN11 knockout populations, as opposed to the depletion of ARID1B and DDX39B knockout populations in a mixed culture of SNU5-Cas9 and knockout populations under both treatments (Fig. 5H–I). However, the knockout of the genes that altered cell death or survival rates under either SN-38 or the SN-38/erlotinib combination, but not both, failed to validate the screen data (Fig. S5C–D). The high correlation between experiment groups treated with SN-38 alone and the SN-38/erlotinib combination and validation experiments suggested remarkably similar genetic dependency/vulnerability signatures for these treatments, indicating shared mechanisms of action.

### Dissecting the mechanism of synergism with RNAi-based signature assay

**Fig. 6:**
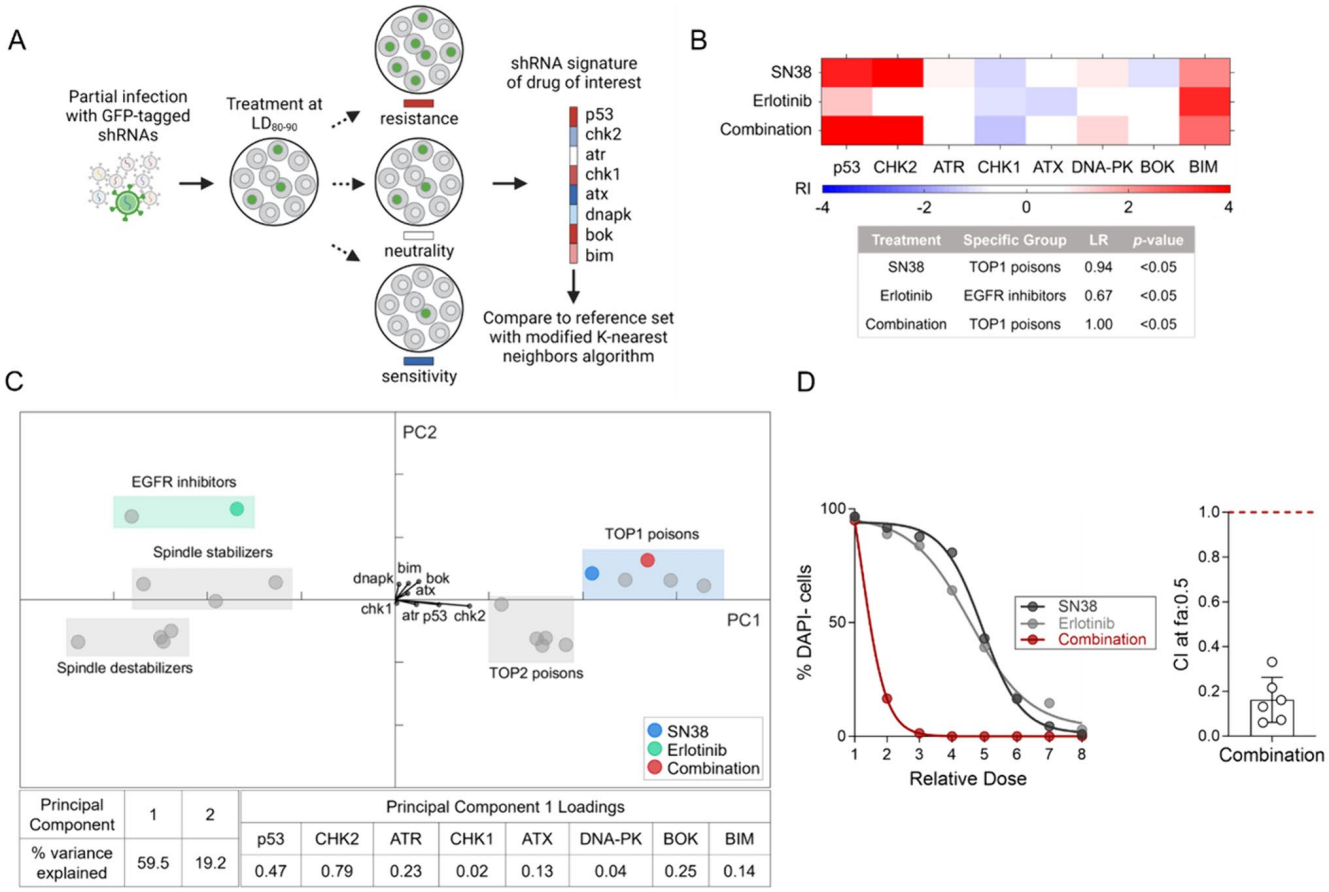
Investigating the mechanism of synergism with the shRNA-based signature assay. **A** Illustration of RNAi-based signature assay, adapted from Jiang et al. [7], using BioRender. LD_80-90_: lethal dose inducing 80–90% cell death. **B** The heatmap generated by assembling resistance index (RI) values for each cell population expressing the specified shRNA shows the signatures of the single agents and SN-38/erlotinib combination (top). Each drug signature was compared with the reference set using the modified K-nearest neighbors algorithm that reports the linkage ratio (LR) and *p* value (bottom). **C** Principal component analysis of TOP1 and TOP2A poisons, EGFR inhibitors, spindle stabilizers, and destabilizers. Boxes in the graph highlight the estimated spatial position of each category within the PCA. The percent variance explained by each principal component and principal component 1 loadings showing each shRNA’s contribution to the analysis is provided in the table. **D** Drug response analysis of the SN-38/erlotinib combination and single agents in Eμ-Myc Cdkn2a^Arf−/−^ cells (left), and the calculation of CI at fa:0.5 for the SN-38/erlotinib combination (right)

To validate whether the SN-38/erlotinib combination employs the exact mechanisms of action with SN-38 alone, we conducted an RNAi-based signature assay, as it provides both statistical and biological generalization for the actions of anti-cancer agents [5, 7, 22]. For this, we generated the signatures of the SN-38/erlotinib combination and each single-agent treatment by assembling resistance index (RI) values for each cell population expressing one of the eight shRNAs. Then we compared the signatures to a reference set using the modified K-nearest neighbors algorithm (Fig. 6A, Fig. S6A). Our results demonstrated that the SN-38/erlotinib combination, like SN-38, clustered in the TOP1 poison category, with a linkage ratio of 1 and *p *value < 0.05 (Fig. 6B, Fig. S6B–C). Thus, this analysis further confirmed that the SN-38/erlotinib combination operated through the exact mechanisms of action of SN-38, as indicated by both biological and statistical classifications.

We performed principal component analysis to examine the variance in our data in fewer dimensions (Fig. 6C), showing the separation of EGFR inhibitors and TOP1 poisons along the first principal component (PC1). Among the original variables from which PC1 was established, shCHK2 strongly contributed to PC1 compared to other hairpins. CHK2 is one of the crucial regulators of the signaling cascade that conveys the DNA damage signal to various downstream effectors [37]. Therefore, this data indicated that the SN-38/erlotinib combination induced DNA damage response, like other members of the TOP1 and TOP2A poisons categories, supporting our previous data in Fig. 3B. We also confirmed a strong synergism between SN-38 and erlotinib in Eμ-Myc Cdkn2a^Arf−/−^ leukemia cells, used as a cell model in the signature assay (Fig. 6D, Fig. S6E–F).

### Exploring the off-target effects of erlotinib

**Fig. 7:**
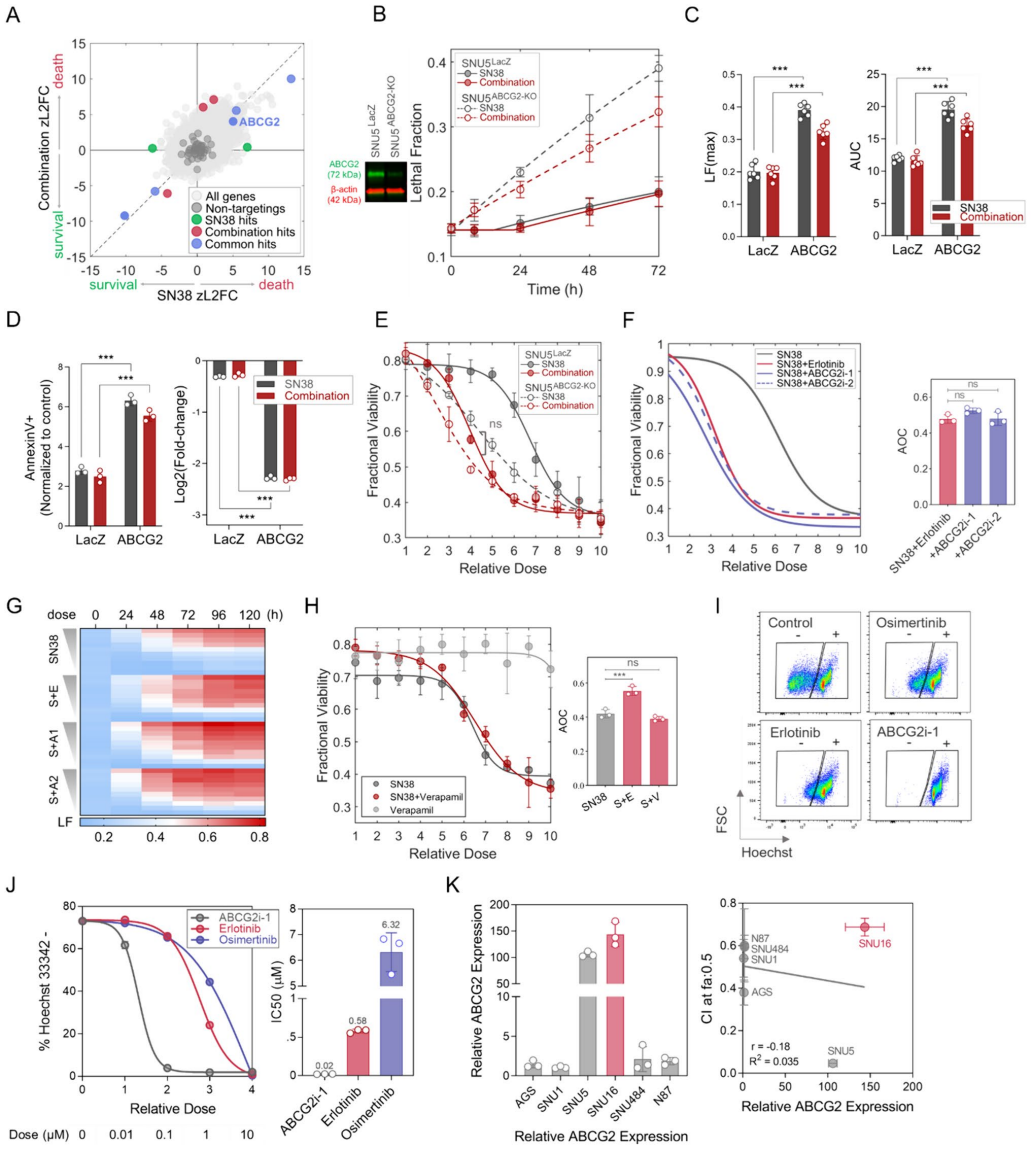
The erlotinib’s off-target effect on ABCG2 is responsible for synergism with SN-38. **A** The screen result shows an enhanced cell death rate under SN-38 and the SN-38/erlotinib combination with the knockout of ABCG2. **B** Analysis of cell death kinetics via lethal fraction (LF) and **C** LF_max_ plots derived from (**B**). Immunoblot in B (left) shows decreased ABCG2 expression in the SNU5^ABCG2−KO^ bulk population compared to SNU5^LacZ^. **D** Annexin V positivity (left) and survival ability assessed by competition assay (right) in SNU5^ABCG2−KO^ compared to SNU5^LacZ^ cells under SN-38 alone and the SN-38/erlotinib combination. **E** Evaluating drug response to SN-38 alone and the SN-38/erlotinib combination in SNU5^ABCG2−KO^ and SNU5^LacZ^ cells using fractional viability (FV). **F–G** Dual combination of SN-38 with ABGC2 inhibitors (ABCG2i-1 and ABCG2i-2) or with erlotinib. **F** Drug response analysis using FV metric (left) and the comparison of AOC values deduced from FV curves (right) in SNU5 cells. **G** Dose-dependent cell death kinetics over time. A1: ABCG2i-1, A2: ABCG2i-2. **H** FV in response to the SN-38/verapamil combination (left), comparison of AOC values (right). *S* SN-38, *E* erlotinib, *V* verapamil. **I–J** Assessment of intracellular Hoechst-33342 accumulation in a dose- and time-dependent manner under erlotinib, osimertinib, or ABCG2i-1 treatments compared to the control. **I** Representative flow plots (1 h). **J** Dose-dependent Hoechst-33342 negativity at the end of the 16-h efflux period (left) and IC_50_ values (right). **K** Relative ABCG2 expression in six gastric cancer cell lines (left) and its correlation with CI at fa: 0.5 values for the SN-38/erlotinib combination (right). Welch’s *t* test was used for all the panels with statistical testing. ***p value < 0.001. *ns* non-significant

Given that EGFR or other RTKs are not involved in the synergistic interaction of erlotinib with SN-38, and the SN-38/erlotinib combination exhibited the same genetic dependency signature as SN-38, we reviewed the literature for other targets of EGFR inhibitors that can potentiate the action of SN-38. Previous studies reported several efflux pumps as off-targets of EGFR inhibitors [38]; hence, we reassessed our genome-wide screening data to find whether the knockout of any efflux pump altered the cell death or survival rate under SN-38 only and combination treatments. We observed that the genetic ablation of ABCG2, also known as breast cancer resistance protein (BCRP) [39], enhanced the cell death rate in the screen under both treatments (Fig. 7A).

By adopting different validation approaches, we demonstrated that knocking out the ABCG2 gene enhanced the drug-induced cell death and impaired the survival ability under SN-38 and the SN-38/erlotinib combination, which confirmed the screen finding (Fig. 7B-D, Figure S7A). Moreover, SN-38-only treatment in SNU5^ABCG2−KO^ cells phenocopied the drug response to the SN-38/erlotinib combination in SNU5^LacZ^ cells (Fig. 7E). Next, we investigated the drug response to the dual combination of SN-38 with different ABCG2 inhibitors, ABCG2i-1 (KO143) and ABCG2i-2 (KS176), in SNU5 cells. The combination of SN-38 with either ABCG2i-1 or ABCG2i-2 (Fig. 7F–G, Fig. S7B–C) achieved a similar degree of drug response and cell death to the SN-38/erlotinib combination. However, the ABCB1 inhibitor verapamil could not increase the response to SN-38 (Fig. 7H). Hence, these results suggested that the synergism in the SN-38/erlotinib combination exhibited a specific dependence on the inhibition of ABCG2 efflux pump activity by erlotinib.

We then investigated the effect of erlotinib on the ABCG2 efflux pump activity in a dose- and time-dependent manner by analyzing the intracellular accumulation of Hoechst-33342 dye, a substrate for ABCG2 (Fig. 7I–J, Fig. S7D–E). We used ABCG2i-1 as the positive control. Since the drug response and immunoblot findings suggested osimertinib as the only EGFR inhibitor with action on RTK activity and no synergism with SN-38 in SNU5 cells (Fig. 4D-I), we employed osimertinib as a negative control treatment. Erlotinib inhibited the ABCG2 efflux activity, but its effect was not as strong as ABCG2i-1, as expected (Fig. 7I–J, Fig. S7D–E). Osimertinib also had an inhibitory effect on the efflux pump activity at high concentrations. However, nearly an 11-fold higher dose of osimertinib was required to achieve the same degree of ABCG2 inhibition as erlotinib. These findings proved that erlotinib was a potent inhibitor of the ABCG2 efflux pump in SNU5 cells, explaining why the SN-38/osimertinib pair failed to induce a strong synergism compared to the SN-38/erlotinib combination. Moreover, the degree of synergism positively correlated with the ABCG2 expression level of gastric adenocarcinoma cell lines, except for SNU16 cells (Fig. 7K).

As supported by our perturbation screen (Fig. 5F, G, and I) and previous studies, decreased SLFN11 expression is responsible for drug resistance to DNA-damaging agents [40]. Hence, this data together suggests that the breach of the correlation between ABCG2 expression and synergy by SNU16 cells can be due to the very low SLFN11 expression in SNU16 cells compared to SNU5 cells (Fig. S7F). SLFN11 is detected as one of the top predictors of response to SN-38 by the DepMap initiative [14, 15], with low SLFN11 expression being associated with a decreased sensitivity to SN-38 in 411 pan-cancer cell models (Fig. S8A–B). Among these cell lines, the ones with high SLFN11 exhibited significantly increased sensitivity to SN-38 (IC50: ~ 10^–6.72^ µM) compared to the ones with low SLFN11 expression (IC50: 10^–5.59^ µM) (Fig. S8C). Furthermore, in cells with high SLFN11 expression, low ABCG2 expression was associated with even lower IC50 values (~ 10^–7.45^ µM) and higher sensitivity to SN-38 (Fig. S8D), while cells with high ABCG2 expression had higher IC50 values (10^–6.59^ µM). Similar to the results in cell models, low SLFN11 expression was associated with poor response to irinotecan in colorectal cancer patients (Fig. S8E–F), the response rates being high in colorectal cancers with high SLFN11 and low ABCG2 expression and low in colorectal cancers with low SLFN11 and high ABCG2 expression (Fig. S8G). All these data supported our findings in gastric adenocarcinoma cells that inhibiting ABCG2 activity in cancers with high SLFN11 expression can increase the efficacy of SN-38. These findings also explain the correlation between the synergy of erlotinib/SN-38 combination with the ABCG2 expression in gastric cancer cells with high SLFN11 expression. To understand whether this correlation is also valid in other cancers, we investigated the correlation between the CI of EGFR inhibitor/SN-38 combination with ABCG2 expression in colorectal cancer cells using data reported in the literature [24, 25]. We observed that the synergy for the gefitinib/SN-38 combination in SLFN11 positive colorectal cancer cells is correlated with the ABCG2 expression (Fig. S8H). These findings suggested that the expression level of ABCG2 could serve as one of the biomarkers for predicting sensitivity to the SN-38/erlotinib combination in cancer cells that are SLFN11 positive and inherently responsive to SN-38.

## Discussion

The paradigm of systemic cancer therapy relies heavily on the strategic combination of anti-cancer drugs with non-overlapping mechanisms and toxicities. This approach aims to increase anti-cancer efficacy, mitigate adverse effects, address tumor heterogeneity, and prevent chemoresistance [3]. The realization of these objectives requires a comprehensive understanding of the mechanistic underpinnings of individual drugs and their combinations, necessitating a meticulous dissection of the genetic dependencies governing pharmacological interactions [4]. While considerable strides have been made in elucidating these facets of conventional chemotherapies [5, 6], the intricate interplay between molecular-targeted agents and chemotherapeutics is yet to be discovered. In this study, we aimed to address this gap by conducting a pairwise screening of molecular-targeted agents with conventional chemotherapeutics in gastric adenocarcinoma models and employing functional genomics methods to offer a rational approach for combination therapies in this challenging cancer.

Our pairwise pharmacological screen identified the SN-38/erlotinib combination as remarkably synergistic in gastric adenocarcinoma cells, surpassing the currently available combination regimens in gastric cancer. This robust synergy, confirmed across different assays and relied on an enhanced cell death rate, prompted further investigation into the underlying genetic signature. Surprisingly, the synergism between SN-38 and erlotinib did not depend on the erlotinib’s primary target, EGFR, leading us to employ genome-wide CRISPR screening and shRNA-based signature assay. Such an approach is also indispensable to identify new and robust targets amenable to inhibition by currently available agents.

Our functional genomics approaches integrated with multistep validation assays consistently demonstrated that the genetic dependency signature of the SN-38/erlotinib combination mirrored that of SN-38 alone. Although the independence of synergism from EGFR increased the possibility of this result, it was still surprising since EGFR inhibitors inhibit several other RTKs in cancer cells [41], which would reprogram intracellular machinery, leading to distinctive dependency signatures.

To uncover the critical players for synergism in the SN-38/erlotinib combination, we revisited our screen data for genes, targeting which may enhance the action of SN-38 without changing the genetic dependency signature. Pharmacological knowledge of cancer chemoresistance indicates that drug efflux pumps can serve such functions. Accordingly, emerging evidence supports that the ABC transporter superfamily can be off-targets of EGFR inhibitors in cancer [38]. Among the ABC transporters, the ABCG2 emerged as a hit enriched in the dead population in both SN-38 monotherapy and SN-38/erlotinib combination groups in our CRISPR screen. Our gene knockout and pharmacological inhibition studies also confirmed the off-target inhibition of ABCG2 by erlotinib as the mechanism of synergism with SN-38 in SNU5 cells, derived from a gastric adenocarcinoma patient who previously received chemotherapy. A previous study showed that erlotinib enhances the ATPase activity of ABCG2 and directly inhibits the pump in a dose-dependent manner [42]. Accordingly, erlotinib increased the accumulation of ABCG2 substrate Hoechst-33342 in our gastric adenocarcinoma cells, similar to the ABCG2 inhibitors. Therefore, our findings, considering previous studies, indicate the direct inhibition of ABCG2 by erlotinib and, hence, decreased efflux of SN-38 as the molecular mechanism of synergy for the erlotinib/SN-38 pair in SNU5 cells.

Given the role of ABCG2 in the efflux of a broad range of chemotherapeutics, translating ABCG2 inhibitors into the clinic is an attractive strategy in cancer [39]. While some ABCG2 inhibitors have been tested in clinical trials [43–45], tyrosine kinase inhibitors are also emerging as ABCG2 inhibitors [38]. Therefore, our findings suggest that combining ABCG2 inhibitors or EGFR inhibitors with SN-38 or irinotecan could be a promising strategy in gastric adenocarcinoma. This strategy may significantly reduce tumor size, especially in ABCG2-positive tumors in the first-line setting. Moreover, in the second-line setting, it may be efficacious to kill clones that become resistant after first-line chemotherapy due to the upregulation of ABCG2. Furthermore, EGFR inhibitors may provide an advantage in highly heterogeneous gastric adenocarcinomas by acting both on cells susceptible to the action on EGFR and cells sensitive to the off-target activity on ABCG2.

The combination of erlotinib with irinotecan or its active metabolite SN-38 holds potential not only for gastric cancer but also for other malignancies. Irinotecan is already a cornerstone in clinical combination protocols for various cancers, including the FOLFIRI (folinic acid/ fluorouracil/ irinotecan) and FOLFOXIRI (folinic acid/ fluorouracil/ oxaliplatin/ irinotecan) regimens for colorectal cancer [46], FOLFIRINOX for pancreatic cancer [47], and the irinotecan–cisplatin regimen for small-cell lung carcinoma [48]. In addition, anti-EGFR strategies are being integrated into combination regimens with conventional chemotherapeutics, especially in colorectal cancer and lung carcinoma [46, 49, 50]. The combination of the anti-EGFR antibody cetuximab with irinotecan or the FOLFIRI regimen has improved treatment outcomes in RAS and BRAF wild-type metastatic colorectal cancers [46]. Consequently, the interest in incorporating EGFR inhibitors like erlotinib into irinotecan-based regimens is increased in metastatic colorectal cancer and potentially increasing in other cancers.

In several xenograft models of colorectal cancer, non-small-cell lung carcinoma, and pancreatic cancer, erlotinib or lapatinib significantly enhanced the anti-tumor efficacy of irinotecan without increasing its toxicity [51–53]. Similarly, an ABCG2 inhibitor also increased the efficacy of irinotecan without increasing toxicity in mouse xenograft models expressing ABCG2 [54]. Several clinical studies also reported promising results. For instance, combining erlotinib with irinotecan and capecitabine was deemed safe enough to warrant further clinical investigation in advanced colorectal cancer patients [55]. Another Phase II study suggested that the combination of erlotinib with irinotecan and the anti-EGFR antibody panitumumab might offer improved efficacy and a good safety profile in metastatic colorectal cancer patients, meriting further investigation [56].

Despite the promising results from in vivo and clinical studies in several cancers, the SN-38/erlotinib combination carries a potential risk of increased adverse effects, since erlotinib also interacts with SN-38 pharmacokinetically at the level of metabolism, increasing plasma concentrations of SN-38 [57]. This may increase the dose-limiting toxicities of SN-38. ABCG2 inhibitors are also prone to a similar pharmacokinetic interaction with SN-38. Another potential impediment also arises from the comparatively low potency of erlotinib in contrast to SN-38, requiring high doses of erlotinib to achieve synergism, thereby posing a limitation to its clinical applicability.

Over the years, liposomal formulations of irinotecan have been developed to enhance its pharmacokinetics. Nanoliposomal irinotecan (nal-IRI) has shown improved anti-tumor efficacy, pharmacokinetics, and safety profiles in xenograft models and has been clinically tested in several cancers, including gastric, colorectal, and pancreatic cancers, leading to its approval for metastatic pancreatic cancer [58]. Recently, a liposomal irinotecan combined with an anti-EGFR/FAP bispecific antibody demonstrated improved efficacy in pancreatic cancer xenograft models [59]. Developing such liposomal irinotecan formulations with EGFR or ABCG2 inhibitors could be a safer approach to achieving synergy with irinotecan in gastric cancer and other gastrointestinal cancers. Therefore, in our future translational studies, we will focus on developing and implementing such nanoliposomal formulations to avoid possible pharmacokinetic interactions and toxicities in combination therapy.

In our screen, erlotinib exhibited synergistic interactions not only with SN-38 but also with the topoisomerase II inhibitor doxorubicin. The degree of synergism for the erlotinib/doxorubicin combination in AGS, SNU1, SNU5, and SNU16 cells paralleled that of erlotinib/SN-38, with the strongest effect observed in SNU5 cells (Fig. 1B). Given that doxorubicin is also a substrate of ABCG2 [60], the inhibition of ABCG2 and the resultant decreased efflux of doxorubicin likely underlie the observed synergism, similar to the interaction seen with the EGFR inhibitor gefitinib and doxorubicin in thyroid cancer [61]. In contrast, the combination indexes for other drug pairs in our panel varied significantly from those of erlotinib/SN-38 and erlotinib/doxorubicin, indicating distinct interaction mechanisms. This antagonistic, additive or weakly synergistic interactions do not provide robust models for strong synergism and are unlikely or less likely to be effective in cancer therapy. Consequently, we have not pursued further investigation into the molecular mechanisms of these interactions.

Our study underscores the strength of functional genomics in uncovering biologically relevant mechanisms of drug action and synergistic interactions in combination therapy. Employing diverse functional genomics approaches that utilize different modes of genetic ablation and a substantially different extent of targeted genes [7, 62], we consistently demonstrated that the genetic signature of the SN-38/erlotinib combination aligns with that of SN-38 alone. Furthermore, we validated the synergistic interaction in cell death and the functional significance of the hit genes in the genetic signature using the FLICK assay [17] and competition assays as powerful tools. Thus, we reliably inferred the mechanism of synergism of a promising drug pair in gastric adenocarcinoma.

From a clinical standpoint, our study suggests that inhibition of ABCG2 to enhance the action of SN-38 can be an effective strategy in tumors with high ABCG2 expression and inherent sensitivity to the DNA-damaging action of SN-38. Our study also challenges the notion that primary drug targets suffice to predict synergism. Molecular-targeted agents are designed to target tumor-specific biomarkers with protumorigenic effect. Since the mutations in the target protein or the downstream signaling pathway are associated with the development of resistance to these agents, preclinical, translational, and precision medicine efforts are directed to catalog such mutations and use this knowledge for patient stratification [63]. However, the insufficiency of the primary drug targets as predictors, coupled with the prominence of off-target dependencies, highlights the importance of assessing both on-target and off-target status for precision medicine strategies. The precision medicine approaches should advance with the evidence indicating off-targets as the main dependencies for the action of numerous molecular-targeted agents [9]. Accordingly, our findings elucidate that proteins that are considered off-target can better predict response to combination therapy in cancers with specific genetic backgrounds. Therefore, a comprehensive understanding of the genetic dependency/vulnerability signatures and cell type-specific mutational landscape is crucial for a more accurate assessment of response to the molecular-targeted agent–conventional chemotherapeutic combinations. Our results support that the ABCG2 status should be assessed in addition to the EGFR and SLFN11 status to improve patient selection strategies for EGFR inhibitor/topoisomerase inhibitor combinations.

In conclusion, our functional genomics approach elucidated that synergistic molecular-targeted agents can act by enhancing the action of conventional chemotherapeutics, leading to an identical genetic dependency/vulnerability profile in combination therapy as conventional chemotherapeutics. The emergence of an off-target, but not on-target, as the predictor of synergism implicates the significance of assessing the off-target status together with the on-target for a more precise selection of patient groups in clinical studies. Further exploration of the interaction between molecular-targeted agent–conventional chemotherapeutic interactions holds promise for developing effective combination regimens in gastric adenocarcinoma, offering potential breakthroughs in a challenging landscape with limited anti-cancer agents. These efforts may also unveil new targeted therapies inducing synergism with conventional chemotherapeutics, presenting opportunities for therapeutic innovation.

## Supplementary Information

Below is the link to the electronic supplementary material.

Supplementary file1 (PDF 2242 KB)

## Data Availability

The data generated in this study are available in the manuscript and its supplementary data. Additional data are available upon request from the corresponding author.
