# Supplementary material for: Functional genomics reveals an off-target dependency of drug synergy in gastric cancer therapy
Source: Gastric Cancer. 2024 Jul 20;27(6):1201–19. doi: 10.1007/s10120-024-01537-y (PMC11513712; doi:10.1007/s10120-024-01537-y)
Supplement: Supplementary file 1 — Supplementary file1 (PDF 2242 KB) [file 10120_2024_1537_MOESM1_ESM.pdf]

Functional genomics reveals an off-target dependency of drug synergy in gastric cancer therapy

Supplementary Figures

A

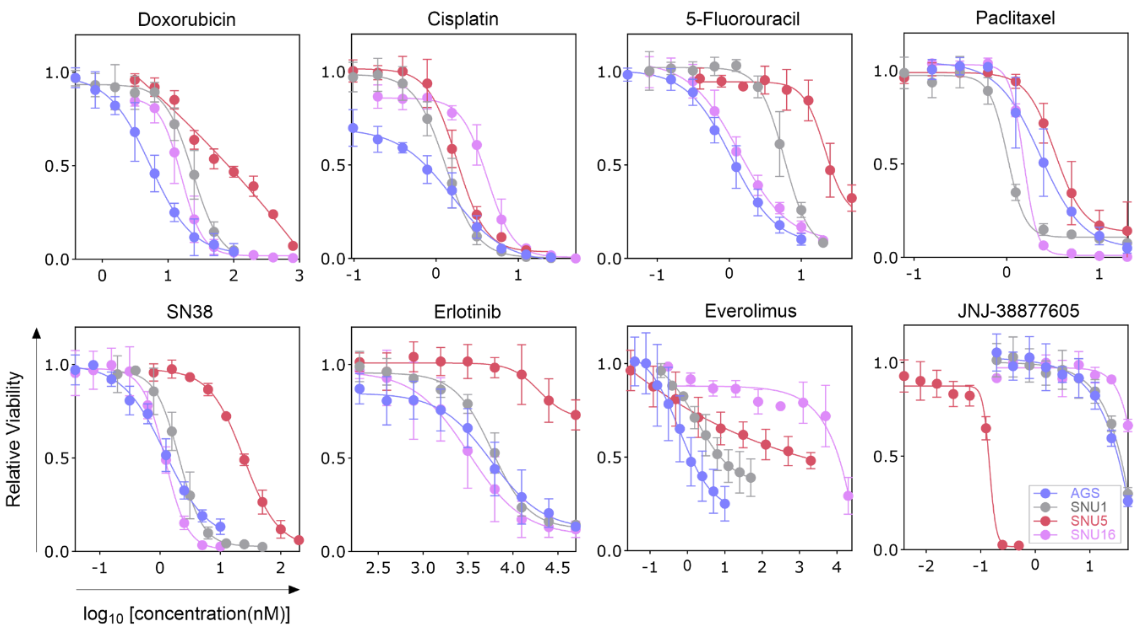

B

Dose \ Cells	AGS	SNU1	SNU5	SNU16	SNU484	N87
1	0.04	0.20	1.95	0.04	0.002	0.02
2	0.08	0.39	3.91	0.08	0.01	0.06
3	0.16	0.78	7.81	0.16	0.02	0.24
4	0.31	1.56	15.6	0.31	0.10	0.98
5	0.63	3.13	31.3	0.63	0.39	3.91
6	1.25	6.25	62.5	1.25	1.56	15.6
7	2.5	12.5	125	2.5	6.25	62.5
8	5	25	250	5	25	250
9	10	50	500	10	100	1000

Dose \ Cells	SNU484 / N87	Others
1	0.195	0.001
2	0.391	0.003
3	0.781	0.012
4	1.563	0.049
5	3.125	0.195
6	6.250	0.781
7	12.50	3.125
8	25	12.5
9	50	50

C D

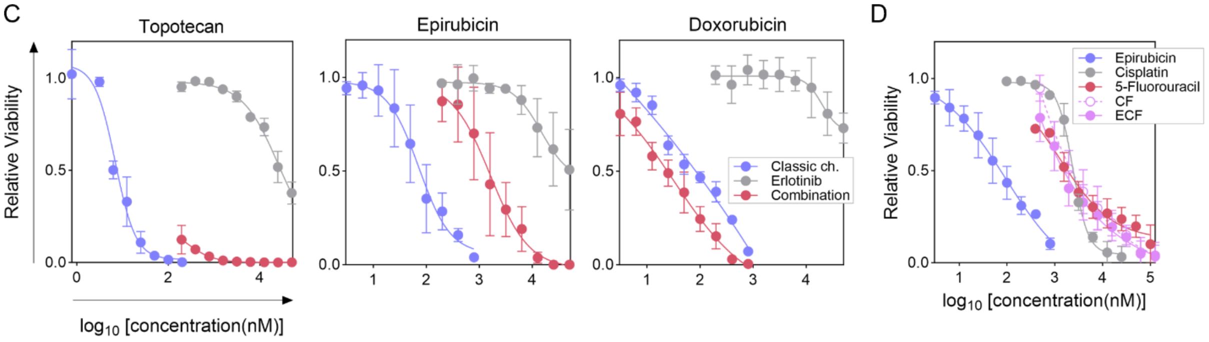

Figure S1. (A) The dose-response curves for conventional chemotherapeutics and molecular-targeted agents in gastric adenocarcinoma cells. **(B)** The relative doses for SN38 in nM (left) and erlotinib in μM (right) used in the combination experiments presented in Figure 1C. **(C)** The dose-relative viability curves for the combination of erlotinib with topotecan, epirubicin, and doxorubicin in SNU5 cells**. (D)** The dose-relative viability curves for epirubicin, cisplatin, 5-fluorouracil, CF (cisplatin and 5-fluorouracil combination), and ECF (epirubicin, cisplatin, 5-fluorouracil) regimen.

A

Dose	SN38 (nM)	Erlotinib (µM)
1	0	0
2	1.95	0.20
3	3.91	0.39
4	7.81	0.78
5	15.6	1.56
6	31.3	3.13
7	62.5	6.25
8	125	12.5
9	250	25
10	500	50

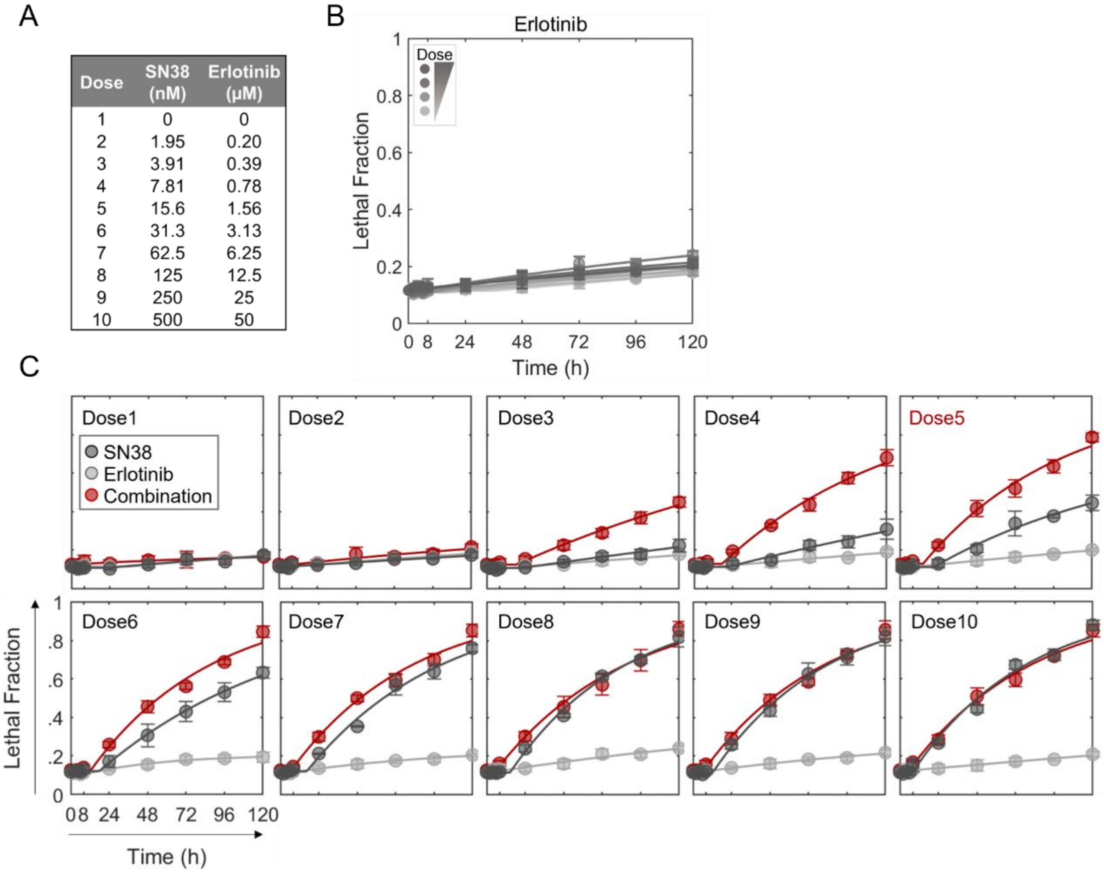

Figure S2. (A) The relative doses of SN38 and erlotinib used in the FLICK assay presented in Figure 2B-D, 4A, and 7E. **(B)** Analysis of the lethal fraction of erlotinib alone for all doses tested. Treatment with erlotinib alone did not substantially induce cell death in SNU5 cells. **(C)** Comparison of lethal fraction curves of the SN38/erlotinib combination and single-agent treatments at each relative dose. Curves at relative dose 5 highlighted are presented in Figure 2E.

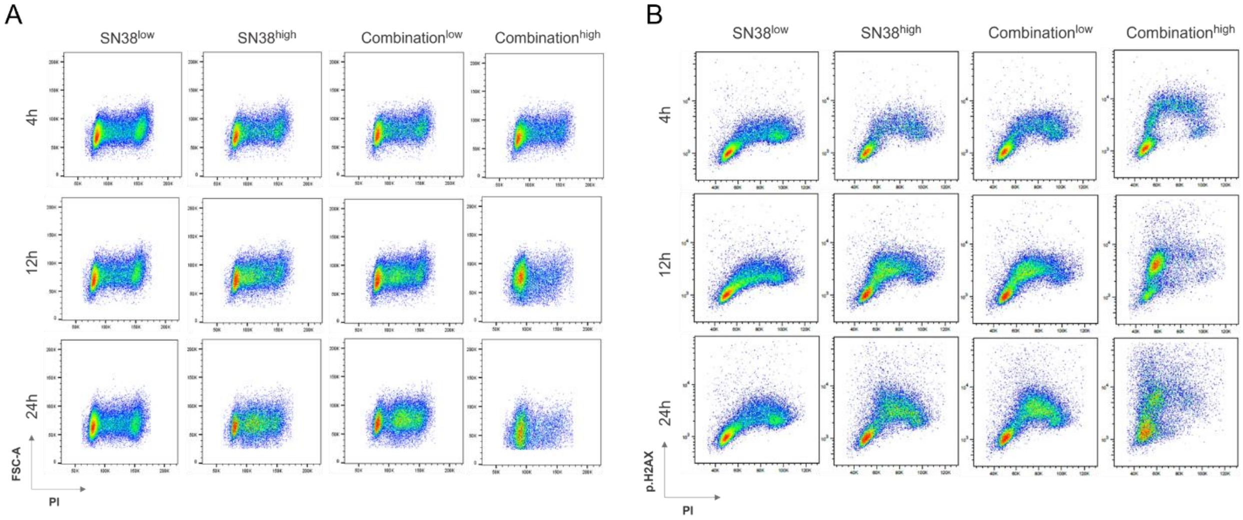

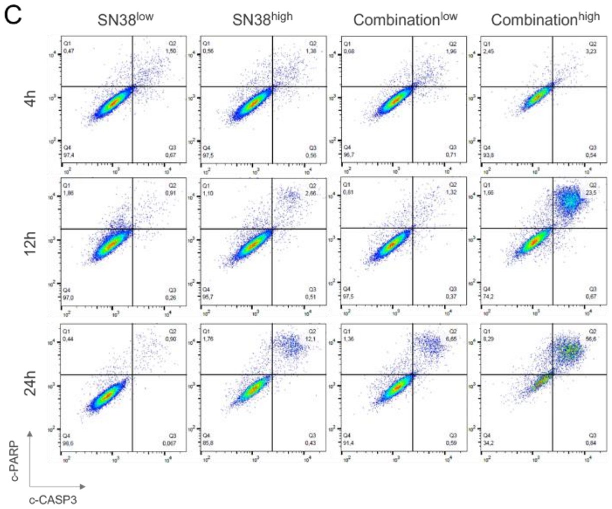

D

SN38^{low}	3.7nM
SN38^{high}	13.5nM
Combination^{low}	3.7nM SN38 + 370nM Erlotinib
Combination^{high}	15.6nM SN38 + 1560nM Erlotinib

Figure S3. Representative flow cytometry plots for **(A)** cell cycle analysis, **(B)** DNA damage profiling coupled with cell cycle analysis, and **(C)** apoptotic cell death analysis in SNU5 cells. c-PARP: cleaved-PARP, c-CASP3: cleaved-caspase3, p.H2AX: phospho-H2AX, PI: propidium iodide. **(D)** Concentrations of erlotinib and SN38 used in (A-C).

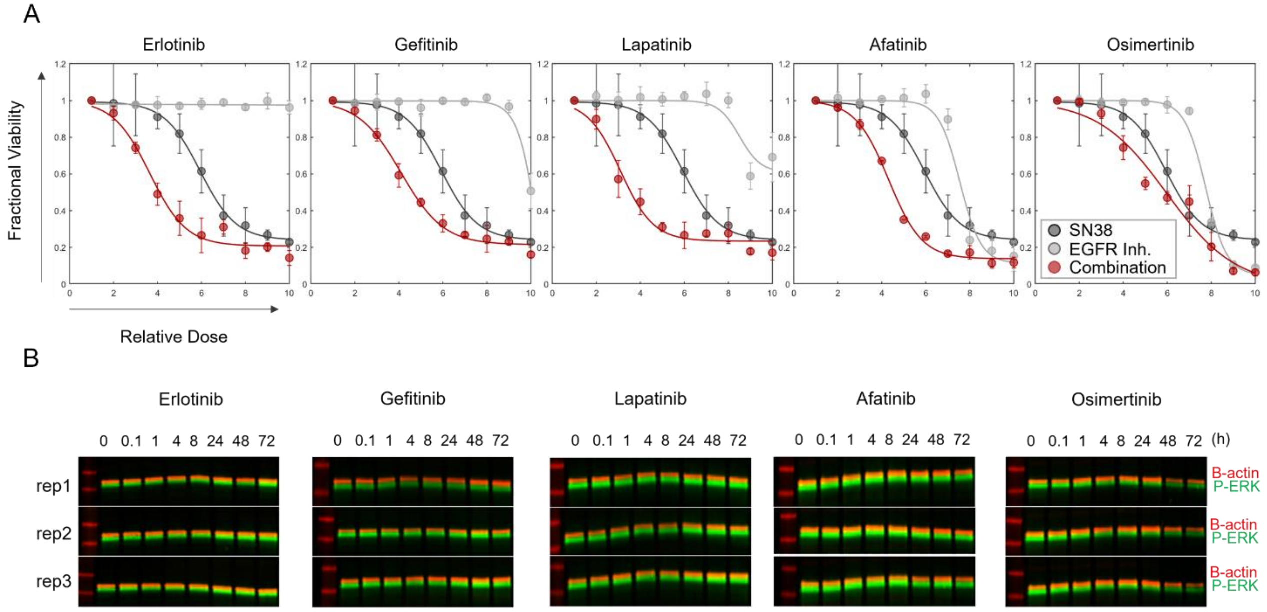

Figure S4. (A) Dose-fractional viability curves for the combination of SN38 with EGFR inhibitors erlotinib, gefitinib, lapatinib, afatinib, and osimertinib. **(B)** The immunoblots (as 3 biological replicates) to assess the effect of EGFR inhibitors on ERK phosphorylation.

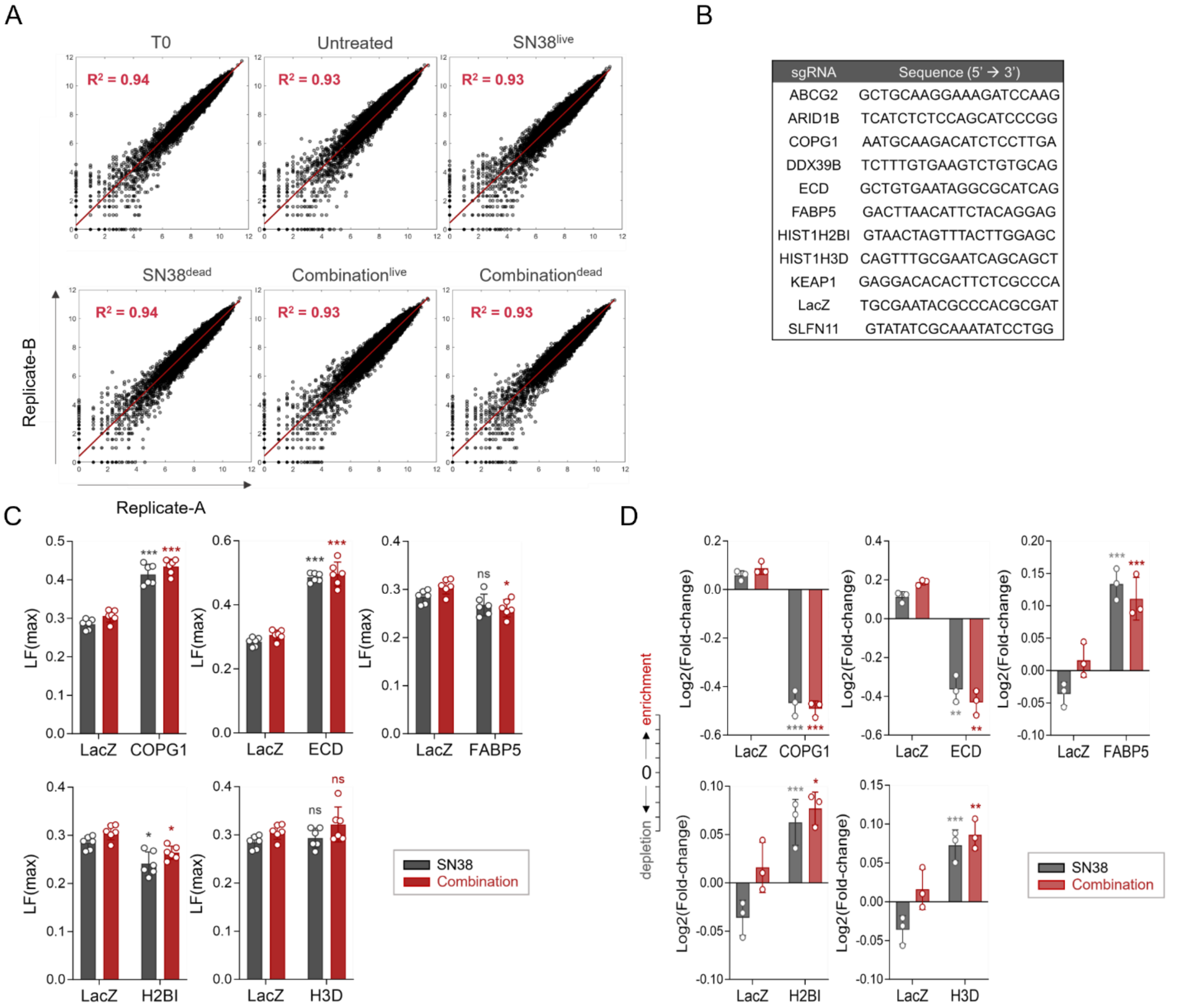

sgRNA	Sequence (5' → 3')
ABCG2	GCTGCAAGGAAAGATCCAAG
ARID1B	TCATCTCTCCAGCATCCCGG
COPG1	AATGCAAGACATCTCCTTGA
DDX39B	TCTTTGTGAAGTCTGTGCAG
ECD	GCTGTGAATAGGCGCATCAG
FABP5	GACTTAACATTCTACAGGAG
HIST1H2BI	GTAACTAGTTTACTTGGAGC
HIST1H3D	CAGTTTGCGAATCAGCAGCT
KEAP1	GAGGACACACTTCTCGCCCA
LacZ	TGCGAATACGCCCACGCGAT
SLFN11	GTATATCGCAAATATCCTGG

Figure S5. (A) Log_2 count level correlation between the biological replicates of each experiment group. x-axis: replicate A at log_2 count level. y-axis: replicate B at log_2 count level. **(B)** sgRNA sequences used to validate the CRISPR screen hits. **(C)** LF (max), and **(D)** Log_2 fold change plots for COPG1, ECD, FABP5, HIST1H2BI (H2BI), or HIST1H3D (H3D) knockout SNU5 cells or untargeted SNU5 cells (LacZ) under SN38 or SN38/erlotinib combination treatment.

A

Category	Drug
TOP2 Poisons	Doxorubicin
	Etoposide
	Epirubicin
	Daunorubicin
	Mitoxantrone
DNA Cross-linkers	Chlorambucil
	Cisplatin
	Satraplatin
	Carboplatin
	Thiotepa
	Mitomycin C
TOP1 Poisons	Camptothecin
	Irinotecan
	SN38
	Topotecan
PARP Inhibitors	BMN-673
	Veliparib
	Olaparib
Single-strand Alkylating Agents	Lomustine
	Carmustine
	Temozolomide
	Streptozocin
Anti-metabolites	Gemcitabine
	Pemetrexed
	Methothrexate
	5-Fluorouracil
	Hydroxyurea
Spindle Destablizers	Vinblastine
	Vinorelbine
	Vincristine
	Colchicine
Spindle Stabilizers	Docetaxel
	Cabazitaxel
	Taxol
Translation Inhibitors	Rapamycin
	Flavopiridol
	BMH-21
	Actinomycin D
HDAC Inhibitors	Scriptaid
	SAHA
	JQ1
	LBH589
DNMT Inhibitors	Zebularine
	5-aza-cytidine
	Decitabine
HSP90 inhibitors	17-AAG
	VER-50589
EGFR Inhibitors	Erlotinib
	Gefitinib

B

	p53	CHK2	ATR	CHK1	ATX	DNAPK	BOK	BIM
SN38	3.534	4.091	0.831	-1.205	0.011	0.969	-1.026	2.144
Erlotinib	1.493	0.313	0.182	-1.017	-1.151	0.547	0.166	3.394
Combination	4.237	4.288	0.626	-1.468	-0.025	1.134	-0.614	2.519

C

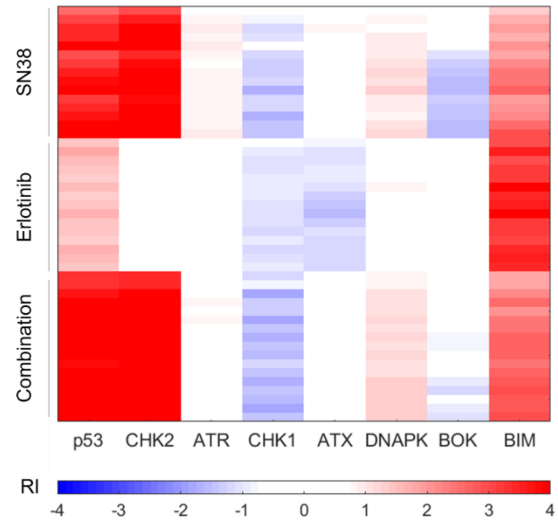

D

shRNA	Target Sequence
p53	CCACTACAAGTACATGTGTAA
CHK2	CAGAAACACATAATCATTAAA
ATR	ACCCATGTTCTTGACATTGAA
CHK1	CAGGAATATTCTGATTGGAAA
ATX	CAGGATAGCAATAAAGATGAA
DNA-PK	CAGGCCTATACTTACAGTTAA
BOK	CTGGCCTCTGTGACTGCTCTA
BIM	TAGGAACAGAGAAATATGCAA

E

Dose	SN38 (nM) / Erlotinib (µM)
1	5
2	7.5
3	10
4	20
5	30
6	40
7	50

F

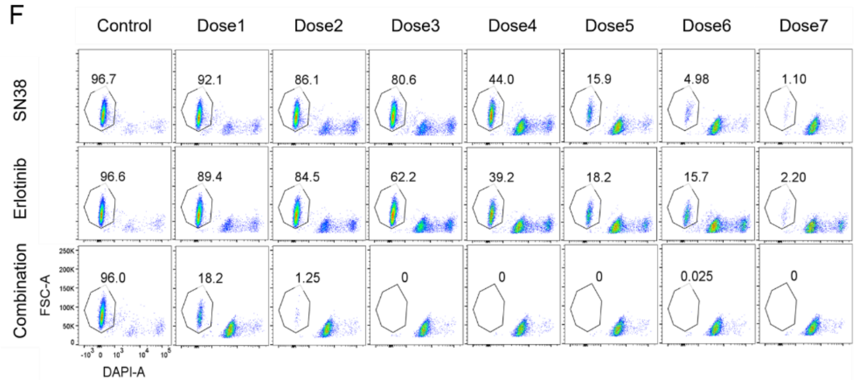

Figure S6. (A) The reference list of drug groups and drugs used to establish shRNA-based signature assay by Pritchard et al. **(B)** Average resistance index (RI) values for each cell population expressing the specified shRNA treated with SN38, erlotinib or SN38/erlotinib combination. **(C)** The heatmap of the signatures for all replicates of each treatment condition generated by assembling the RI values. **(D)** the shRNA sequences used in the signature assay. **(E)** The relative doses of SN38 and erlotinib applied for the dose-response analyses in Eμ-Myc Cdkn2a$^{Arf-/-}$ cells, presented in Figure 6D. **(F)** Representative flow cytometry plots of live cell fractions for each tested dose of SN38, erlotinib, or SN38/erlotinib combination in the signature assay.

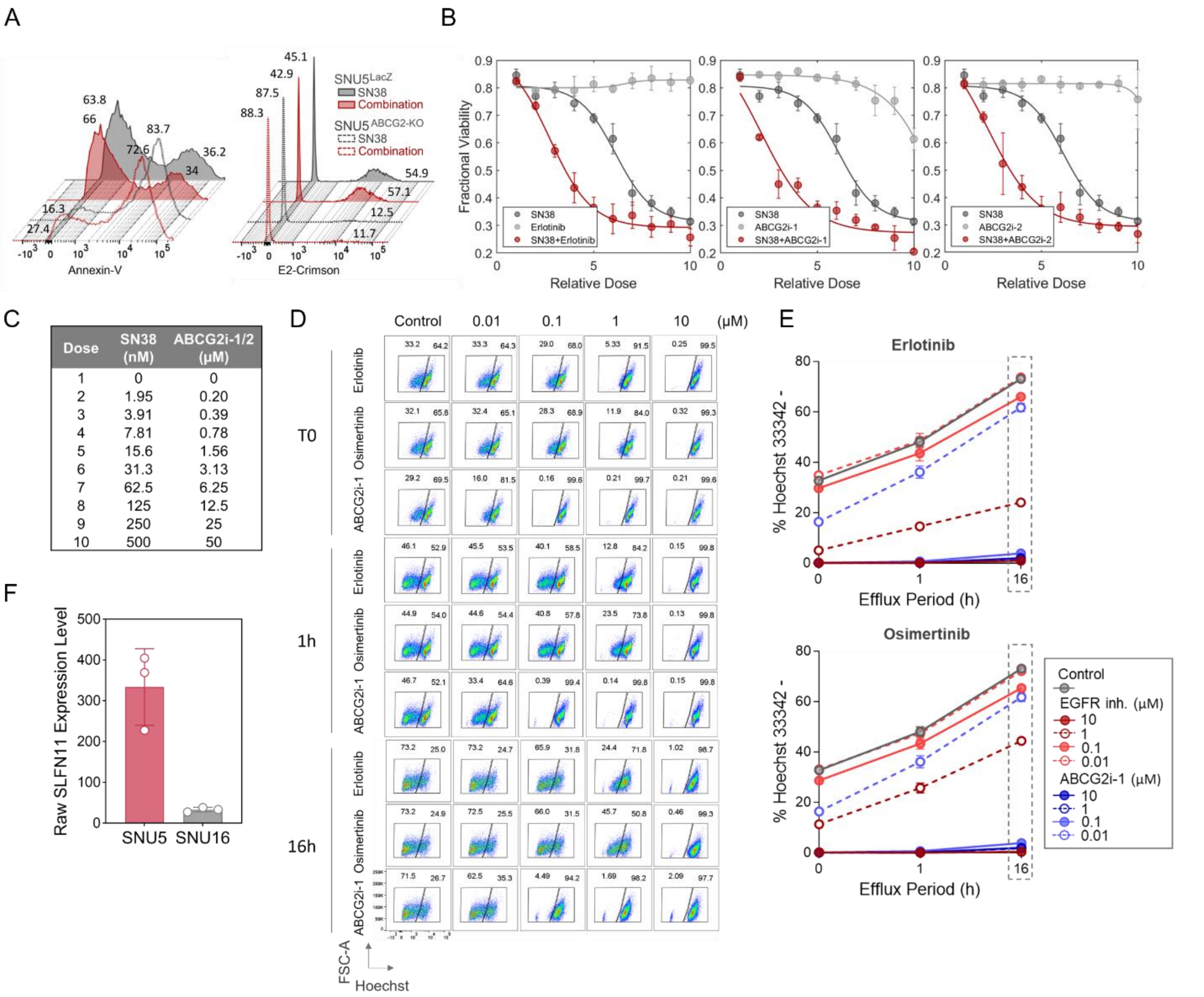

Dose	SN38 (nM)	ABCG2i-1/2 (μM)
1	0	0
2	1.95	0.20
3	3.91	0.39
4	7.81	0.78
5	15.6	1.56
6	31.3	3.13
7	62.5	6.25
8	125	12.5
9	250	25
10	500	50

Figure S7. (A) Representative histogram plots of annexin-V (left) and e2-crimson (right) positivity in SNU5^{LacZ} and SNU5$^{ABCG2\text{-}KO}$ cells treated with SN38 or SN38-erlotinib combination. **(B)** Dose-fractional viability curves for the dual combination of SN38 with erlotinib, ABCG2i-1, or ABCG2i-2 in SNU5 cells. **(C)** The concentrations of SN38 and ABCG2 inhibitors for the curves in B and Figure 7F. **(D)** Assessment of the impact of ABCG2 and EGFR inhibitors on the efflux of Hoechst over time in SNU5 cells via flow cytometry. **(E)** Percent inhibition of Hoechst efflux by ABCG2 and EGFR inhibitors over time in SNU5 cells calculated from the flow cytometry plots in D. **(F)** The gene expression level of SLFN11 in SNU5 and SNU16 cells. Expression data was exported from merav.wi.mit.edu/ (Shaul et al.2015).

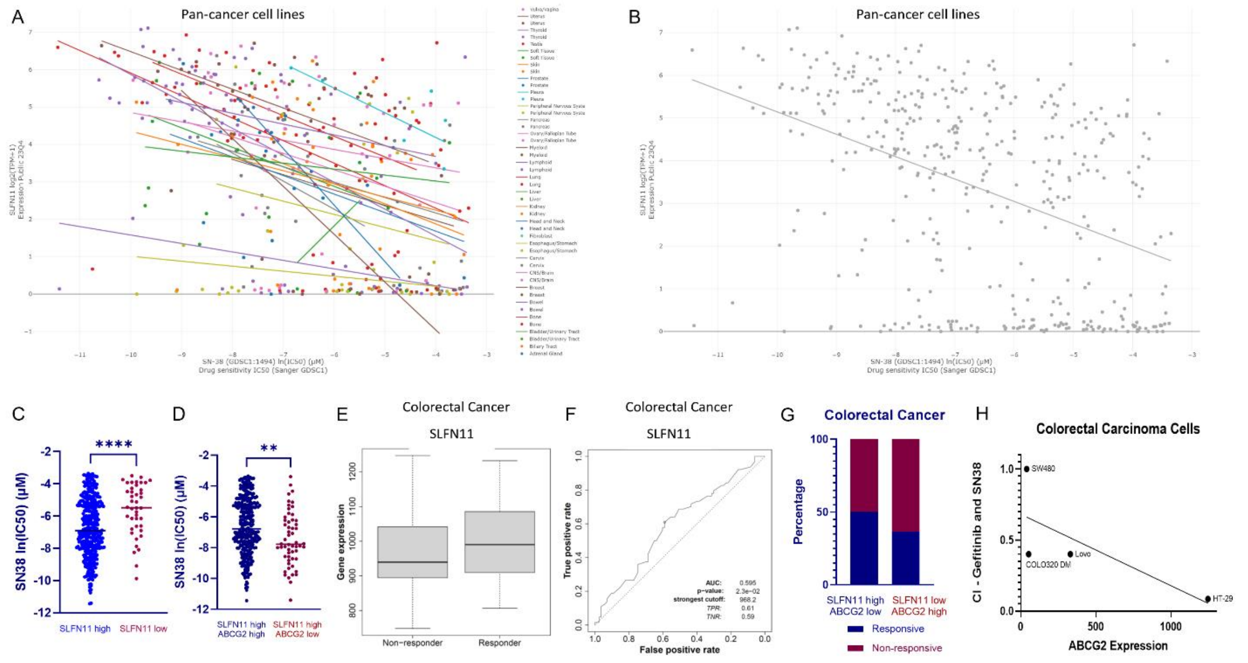

Figure S8. (A) Cell lineage-specific correlation between the expression of SLFN11 and sensitivity to SN-38 in 411 pan-cancer cell lines. **(B)** The general correlation between the expression of SLFN11 and sensitivity to SN-38 in 411 pan-cancer cell lines. (**C**) IC50 of SN38 in cells with high SLFN11 expression vs. low SLFN11 expression. (**D**) IC50 of SN38 in cells with high SLFN11 and high ABCG2 expression vs. high SLFN11 and low ABCG2 expression. (**E**) The expression profile of SLFN11 in colorectal cancer patients responsive vs. non-responsive to irinotecan. (**F**) The ROC plot shows the predictive value of SLFN11 in colorectal cancer patients who were treated with irinotecan. (**G**) The percent distribution of responders vs. non-responders to irinotecan treatment in colorectal cancer patients with high SLFN11 and low ABCG2 expression vs. low SLFN11 and high ABCG2 expression. (**H**) The correlation between ABCG2 expression and combination index (CI) of SN38-gefitinib combination in colorectal carcinoma cell lines with similar SLFN11 expression. In A and B, the graphs generated by DepMap Portal (https://depmap.org/portal/) were downloaded (DepMap Broad, 2019; Ghandi et al. 2019). In C and D, the gene expression and SN-38 IC50 data downloaded from the DepMap Portal were analyzed and used to generate the bar graphs in GraphPad Prism 10. The medians of SLFN11 and ABCG2 expression among 411 cell lines were used as a cut-off to classify cell lines as SLFN11 and ABCG2 high or low. In E and F, the graphs were downloaded from RocPlotter (https://rocplot.org/) (Fekete & Győrffy 2023). In G, data for colorectal cancer patients downloaded from the RocPlotter was analyzed. In H, ABCG2 gene expression levels of cell lines were downloaded from merav.wi.mit.edu/ (Shaul et al.2015). The CI of SN38-gefitinib combination in HT-29 cells was retrieved from Azzariti et al. 2004, and in COLO320 DM, Lovo, and SW480 cells, the CI values were retrieved from Kouzimu et al. 2004. The CI values at fa=0.5 (fraction of affected cells =0.5) were used in the analysis.

References

Azzariti, A., Xu, J. M., Porcelli, L., & Paradiso, A. (2004). The schedule-dependent enhanced cytotoxic activity of 7-ethyl-10-hydroxy-camptothecin (SN-38) in combination with Gefitinib (Iressa, ZD1839). Biochemical pharmacology, 68(1), 135–144. https://doi.org/10.1016/j.bcp.2004.03.014

DepMap, Broad (2019): DepMap 19Q4 Public. figshare. Dataset doi:10.6084/m9.figshare.11384241.v2.

Fekete, J. T., & Győrffy, B. (2023). New Transcriptomic Biomarkers of 5-Fluorouracil Resistance. International journal of molecular sciences, 24(2), 1508. https://doi.org/10.3390/ijms24021508

Ghandi, M., Huang, F. W., Jané-Valbuena, J., Kryukov, G. V., Lo, C. C., McDonald, E. R., 3rd, Barretina, J., Gelfand, E. T., Bielski, C. M., Li, H., Hu, K., Andreev-Drakhlin, A. Y., Kim, J., Hess, J. M., Haas, B. J., Aguet, F., Weir, B. A., Rothberg, M. V., Paolella, B. R., Lawrence, M. S., … Sellers, W. R. (2019). Next-generation characterization of the Cancer Cell Line Encyclopedia. Nature, 569(7757), 503–508. https://doi.org/10.1038/s41586-019-1186-3

Koizumi, F., Kanzawa, F., Ueda, Y., Koh, Y., Tsukiyama, S., Taguchi, F., Tamura, T., Saijo, N., & Nishio, K. (2004). Synergistic interaction between the EGFR tyrosine kinase inhibitor gefitinib ("Iressa") and the DNA topoisomerase I inhibitor CPT-11 (irinotecan) in human colorectal cancer cells. International journal of cancer, 108(3), 464–472. https://doi.org/10.1002/ijc.11539

Shaul, Y.D., Yuan, B., Thiru, P., Nutter-Upham, A., McCallum, S., Lanzkron, C., Bell, G.W., Sabatini, D.M. (2015) MERAV: a tool for comparing gene expression across human tissues and cell types. Nucleic Acids Res. 44 (560-566)
